# Combining single-cell RNA sequencing of peripheral blood mononuclear cells and exosomal transcriptome to reveal the cellular and genetic profiles in COPD

**DOI:** 10.1186/s12931-022-02182-8

**Published:** 2022-09-20

**Authors:** Yanli Pei, Yuxi Wei, Boshizhang Peng, Mengqi Wang, Wei Xu, Zhe Chen, Xindi Ke, Lei Rong

**Affiliations:** 1 Respiratory Medicine Department, The University of Hong Kong-Shenzhen Hospital, Shenzhen, China; 2 Peking Union Medical College (PUMC), PUMC and Chinese Academy of Medical Sciences, Beijing, China; 3 Laboratory of Cough, Affiliated Kunshan Hospital of Jiangsu University, Suzhou, Jiangsu China

**Keywords:** COPD, Immunity, Exosome, Single-cell RNA sequencing

## Abstract

**Background:**

It has been a long-held consensus that immune reactions primarily mediate the pathology of chronic obstructive pulmonary disease (COPD), and that exosomes may participate in immune regulation in COPD. However, the relationship between exosomes and peripheral immune status in patients with COPD remains unclear.

**Methods:**

In this study, we sequenced plasma exosomes and performed single-cell RNA sequencing on peripheral blood mononuclear cells (PBMCs) from patients with COPD and healthy controls. Finally, we constructed competing endogenous RNA (ceRNA) and protein–protein interaction (PPI) networks to delineate the interactions between PBMCs and exosomes within COPD.

**Results:**

We identified 135 mRNAs, 132 lncRNAs, and 359 circRNAs from exosomes that were differentially expressed in six patients with COPD compared with four healthy controls. Functional enrichment analyses revealed that many of these differentially expressed RNAs were involved in immune responses including defending viral infection and cytokine–cytokine receptor interaction. We also identified 18 distinct cell clusters of PBMCs in one patient and one control by using an unsupervised cluster analysis called uniform manifold approximation and projection (UMAP). According to resultant cell identification, it was likely that the proportions of monocytes, dendritic cells, and natural killer cells increased in the COPD patient we tested, meanwhile the proportions of B cells, CD4 + T cells, and naïve CD8 + T cells declined. Notably, CD8 + T effector memory CD45RA + (Temra) cell and CD8 + effector memory T (Tem) cell levels were elevated in patient with COPD, which were marked by their lower capacity to differentiate due to their terminal differentiation state and lower reactive capacity to viral pathogens.

**Conclusions:**

We generated exosomal RNA profiling and single-cell transcriptomic profiling of PBMCs in COPD, described possible connection between impaired immune function and COPD development, and finally determined the possible role of exosomes in mediating local and systemic immune reactions.

**Supplementary Information:**

The online version contains supplementary material available at 10.1186/s12931-022-02182-8.

## Background

As the rate of demographic aging and urbanisation continues to increase, chronic respiratory diseases place increasing health and economic burdens on all nations [1]. A systematic review reported that the global prevalence of chronic obstructive pulmonary disease (COPD) was 10.3% in 2019 according to the GOLD definition, affecting 391.9 million people [2]. Deaths due to COPD accounted for 5.7% of all deaths in 2017 [1]. China, as the most populous country, accounts for most of the global burden of COPD. Based on a nationally representative sample, the estimated prevalence of COPD in China was 13.6%, and COPD was the fifth leading cause of death nationwide in 2016 [3]. However, most existing treatments for COPD aim to relieve symptoms and avoid acute exacerbation. More targeted interventions are still lacking, partially because the pathogenesis of this disease is not well established. Therefore, clarifying the pathophysiology of COPD is a major focus in the field of respiratory diseases.

Patients with COPD suffer from consistent airway limitation, which is predominantly caused by airway inflammation and is characterised by an increased release of pro-inflammatory factors and higher counts of immune cells [4]. COPD itself is a highly heterogeneous disease of complex and diverse origins. Instead of tobacco-stimulated chronic inflammation in genetically susceptible individuals, COPD has recently been recognised as an accumulation of gene-exposure interactions accompanied by aging [5].

The characteristics of the peripheral immune system remain elusive, hence, the regulatory properties of immune reactions in COPD warrants further study. Efforts have been made to identify the distinct inflammatory endotypes underlying COPD based on sputum and blood biomarkers [6]. Moreover, it has been proven that local and peripheral biomarkers, especially immune-related biomarkers, are associated with COPD phenotypes at the cellular and molecular levels. The ability of blood transcriptomes to predict COPD development and progression [7] has also been illustrated, and several gene expression signatures stimulated by interferons have been associated with COPD clinical traits [8]. Gene expression signatures of peripheral blood mononuclear cells (PBMCs), representative of systemic immune function, have been validated to play critical roles in COPD pathogenesis [9]. Nonetheless, the mechanism by which the immune system is regulated during COPD progression remains unknown. Evidence has shown that the activation of immune reactions relies on intercellular mediators, including exosomes [10]. Derived from either lung resident macrophages or bronchial epithelial cells, exosomes can induce the production of cytokines and other signal transduction molecules that activate downstream immune cell recruitment and other inflammatory reactions [11].

Despite the great strides in our knowledge of COPD, the relationship between local airway inflammation and peripheral immune status remains unclear. Considering the robust evidence regarding the association between exosomes, immune cells, and COPD pathophysiology, we aimed to determine the connection between exosomes and peripheral immune patterns in COPD. To illustrate the immune landscape of PBMCs in COPD, we performed deep single-cell RNA (scRNA) sequencing. By visualising protein–protein interactions between exosomal RNA and PBMCs, we intended to identify potential exosomal RNA signatures of inflammation-related signalling transduction and explore the possible roles of immune cells in COPD pathology, shedding light on the mechanisms underlying impaired immune functions in patients with COPD.

## Methods

### Collection of human specimens

Patients with COPD who were hospitalised at the University of Hong Kong-Shenzhen Hospital served as the disease group (n = 6), while healthy volunteers served as the control group (n = 4). Two blood samples per participant for exosome isolation and PBMC preparation respectively and clinical information were collected upon their first day of hospitalization. Baseline characteristics of all the participants are provided in Table 1. One blood sample from each participant was used for exosomal RNA sequencing which data was merged into an RNA expression matrix sorted by group. Two subjects without complications, Dis6 from the disease group and Con4 from the control group were selected for PBMC single-cell RNA sequencing.

**Table 1:** Baseline characteristics of the subjects included

	Dis1	Dis2	Dis3	Dis4	Dis5	Dis6	Con1	Con2	Con3	Con4
Gender	Male	Male	Male	Female	Male	Male	Female	Male	Male	Male
Age	61	60	64	62	38	70	54	59	53	47
BMI (kg/m^2^)	21.0	18.0	19.5	21.4	23.1	18.6	23.9	21.5	21.0	22.0
Smoke^*^	Yes	Yes	Yes	None	Yes	Yes	None	None	None	None
CAT	13	5	6	11	6	20				
sNEU (%)	72.93	95.31	90.37	88.72						
sLYM (%)	2.63	1.27	1.17	1.43						
sMAC (%)	23.3	1.27	5.89	8.39						
sEOS (%)	1.12	2.12	2.55	1.43						
FVC (L/%)	3.76/93.1	3.44/93.2	3.01/82	1.83/75.9	3.81/93					
FEV1 (L/%)	1.88/59	1.5/51.2	1.65/57.2	0.94/46.6	2.62/76					
FEV1/FVC (%)	49.9	43.5	57.2	51.3	68.9					
GOLD	2	2	2	3	2					
bWBC (10^9^/L)	8.30	6.79	7.06	8.47	6.74					
bNEU (%)	64.3	52.6	58.5	68.7	62.5					
bNEU#	5.33	3.57	4.13	5.82	4.21					

*Dis* disease, *Con* control, *CAT* COPD assessment test, *BMI* body mass index, *sNEU* sputum neutrophil, *sLYM* sputum lymphocyte, *sMAC* sputum macrophage, *sEOS* sputum eosinophil, *FVC* forced vital capacity, *FEV1* forced expiratory volume in 1 s, *GOLD* global initiative for chronic obstructive lung disease, *bWBC* blood white blood cell, *bNEU* blood neutrophil

^*^Including former and current smoker

### Exosome isolation

The ethylenediaminetetraacetic acid-anticoagulated blood samples were centrifuged at 10,000 × *g* for 15 min at 4 °C to remove debris. The supernatants were washed with phosphate-buffered saline (PBS) and treated with proteinase K (Life Technologies, MD, USA) for 10 min at 37 °C. Total exosome isolation reagent (Invitrogen, Gaithersburg, MD, USA) was added to the tubes and incubated for 30 min at 4 °C. Subsequently, exosomes were retained in the sediment via centrifugation at 10,000 × *g* for 5 min at 4 °C. After centrifugation under the same conditions for 1 min, the supernatants were removed, and the exosomes were incubated in 110 μL PBS for 3 min at 37 °C. The exosome pellet was resuspended for subsequent experiments.

### Exosome characterisation

Transmission electron microscopy (TEM) (HT-7800, Hitachi High-Technologies Corporation, Minato, Tokyo, Japan) was used to examine the morphology of the isolated exosomes. First, 5 μL of the isolated exosomes were diluted with PBS to a volume of 10 μL before examination. The samples were then dropped onto a copper screen for 1 min, and the excess suspension was removed using filter paper. Subsequently, 10 μL of 2% uranyl acetate was added to the deposited exosomes for 1 min, and the excess suspension was removed. The copper screen was dried for a few minutes at 25 °C, and then TEM images of the isolated exosomes were acquired at 80 kV.

Nano-flow cytometry (nFCM) (Flow Nano-Analyzer, NanoFCM Inc., Xiamen, China) was used to measure the concentration and size of the isolated exosomes. First, isolated exosomes (5 μL) were diluted to 30 μL for examination. After performance testing of the instrument using standards, the exosomes were loaded into the nFCM instrument to determine their concentration and size. Identification of exosome-specific proteins was also performed using nFCM. Briefly, 30 μL of the isolated exosomes were diluted 1:4 and equally distributed into four sterile tubes. Next, 30 μL of each diluted sample was stained with 20 μL fluorescently labelled anti-human IgG (BioLegend, San Diego, CA, USA), anti-CD9, -CD63, and -CD81 antibodies (BD Biosciences, Franklin Lakes, NJ, USA), and incubated at 37 °C for 30 min. Subsequently, the mixture was mixed with 1 mL of precooled PBS and centrifuged at 110,000 × *g* for 70 min at 4 °C, after which the supernatant was removed. The resulting exosome pellet was resuspended in 50 μL precooled PBS for nFCM detection.

### RNA extraction, library construction, and RNA sequencing

Exosomal RNA sequencing was performed at the Beijing Genomics Institute (BGI, Shenzhen, China). Prior to sequencing, RNA was extracted from exosomes using TRIzol reagent (Life Technologies). An Agilent 2100 Bioanalyzer (Thermo Fisher Scientific) and qRT-PCR were used to evaluate the quality and quantity of the extracted RNA to meet the specifications for library generation performance. MGIEasy rRNA depletion kits (MGI, Shenzhen, China) were used to remove the rRNA. The purified RNA was then used to construct a cDNA library using an MGIEasy RNA Directional Library Prep Kit (MGI). The constructed library was sequenced on an Illumina DNBSEQ platform, the raw reads generated were filtered using SOAPnuke software (version 1.5.2) [12] to obtain clean reads, and were saved in FASTQ format. After the clean reads were aligned to the reference genome and transcriptome using Bowtie2 software (version 2.2.5) [13], the mapped reads were used for subsequent analyses. Between disease group and control group, differentially expressed exosomal mRNAs, lncRNAs, and circRNAs were identified using the DESeq2 package [14], based on the criteria |log_2_FC|> 1.0 and adjusted p-value (Q value) < 0.05.

### Competing endogenous RNA (ceRNA) network construction

Bioinformatic databases were applied to construct ceRNA network. We used TargetScan (http://www.targetscan.org/) to predict miRNA targets for differentially expressed mRNAs, miRcode (http://www.mircode.org/) to predict miRNA targets for differentially expressed lncRNAs and starBase (http://starbase.sysu.edu.cn/) to predict miRNA targets for differentially expressed circRNAs identified before. After identifying miRNA targets, all pairs of miRNA and differentially expressed mRNAs/lncRNAs/cirRNAs were retrieved from the websites of these databases by directly downloading (miRcode) or programming script (TargetScan and starBase). Then, mRNA-miRNA pairs which have overlapped miRNA targets with lncRNA-miRNA or circRNA-miRNA pairs were screened. Finally, these mRNA-miRNA pairs together with matched lncRNA-miRNA and circRNA-miRNA pairs were input onto Cytoscape software to construct a ceRNA network.

### Single-cell preparation and sequencing

PBMCs were isolated from another blood samples of Dis6 and Con4 using the Ficoll-Paque Plus reagent (GE Healthcare). The PBMC suspensions were then loaded onto a haemocytometer for cell counting and viability examination. The viability of all the assessed samples exceeded 80%, and the cell densities of the suspensions were adjusted to 700–1200 cells/μL. The suspensions were then loaded onto microfluidic chips from the Single Cell 3′Chip Kit (10 × Genomics, CA, USA) to prepare single-cell gel beads-in-emulsions (GEMs). GEMs were subsequently subjected to cDNA library construction using a Chromium Single Cell 3′ Reagent Kit v2 (10 × Genomics). Libraries were sequenced using the BGISEQ-500 instrument. CellRanger software (version 5.0.1) was used to process the raw reads, map the reads, and quantify gene expression. Seurat software (version 3.0.2) was used for quality control and to identify highly variable genes. Single-cell RNA transcriptome analysis was then performed based on the expression matrices generated from the above steps.

### Unsupervised clustering and cell type annotation

The filtered expression matrices were subjected to unsupervised cell clustering using Seurat software (version 3.0.2). All cells were pooled for dimensional reduction using principal component analysis based on the top 20 significant principal components and the 2000 most variable genes. These pooled cells were clustered with a resolution of 0.5 and visualized using the graph-based visualisation method uniform manifold approximation and projection (UMAP). The expression levels of each cluster were compared with the remaining clusters. Genes with a |log_2_FC|> 0.25 and Q value < 0.05 were identified as marker genes. Clusters were first annotated automatically using the SCSA software and then modified artificially based on the expression of canonical markers of known cell types [15].

### Functional annotation

All differentially expressed genes and marker genes were annotated based on the Gene Ontology (GO) and Kyoto Encyclopedia of Genes (KEGG) databases. GO and KEGG enrichment analyses were carried out using the R function “Phyper”. The GO or KEGG terms that matched the criteria of p-value < 0.05 were considered significantly enriched.

### Statistical analysis

Continuous variables are presented as the mean ± standard deviation. Statistical analyses and visualisations were performed using R software (version 4.0), while the Pearson method was used for the correlation analyses. Unless specified, statistical significance was accepted at a bilateral p-value < 0.05.

## Results

### Isolation and identification of exosomes from plasma

**Fig. 1:**
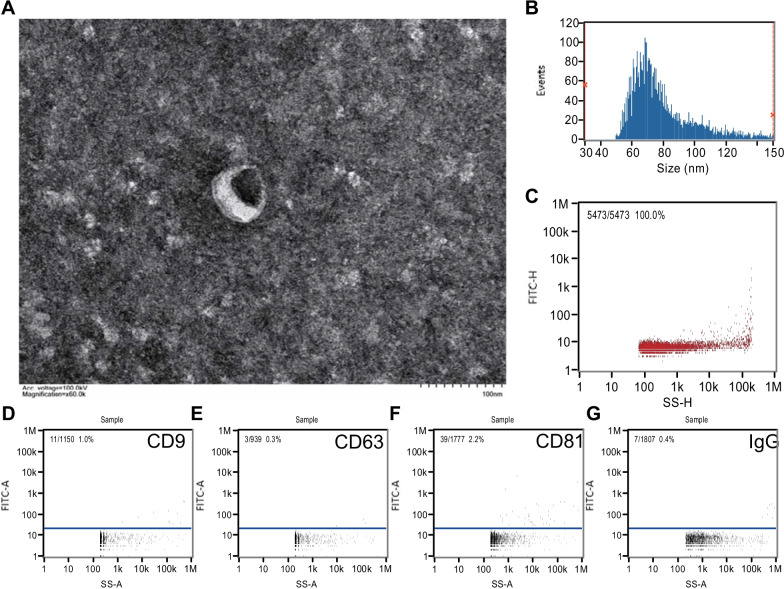
Identification of exosomes from plasma. **A** Electron micrograph of exosomes imaged under transmission electron microscopy (scale bar = 100 nm). **B** Diameter and **C** concentration of exosomes as detected using nanoflow cytometry measurement (nFCM). The expression of surface markers **D** CD9, **E** CD63, **F** CD81, and **G** IgG (as a negative control) was also detected using nFCM

TEM imaging revealed the typical double-layer rounded shape of the vesicles (Fig. 1A). nFCM analysis showed that the particle size was 73.25 ± 20.28 nm (median ± SD) and the average concentration of the particles was 8.17 × 10^8^/mL, verifying that they were within the typical particle size distribution and higher than the detection limit [16] (Fig. 1B, C). Furthermore, the presence of exosomal marker proteins, including CD9, CD63, and CD81, was confirmed through nFCM analysis, where IgG was regarded as the isotype control antibody (Fig. 1D–G). These results indicate that exosomes were successfully extracted from the plasma samples.

### Differentially expressed exosomal mRNAs, lncRNAs, circRNAs, and association between differentially expressed mRNAs and clinical characteristics

**Fig. 2:**
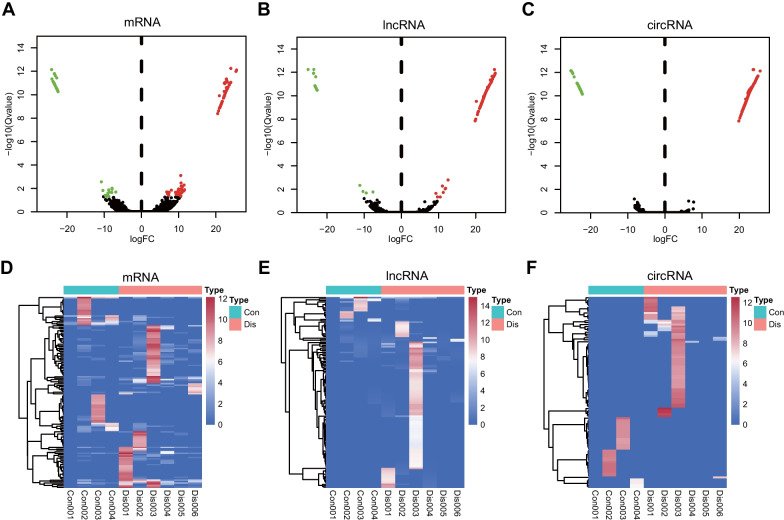
Identification of exosomal differentially expressed mRNAs, lncRNAs, and circRNAs in COPD. Volcano plots of exosomal differentially expressed **A** mRNAs, **B** lncRNAs, and **C** circRNAs. The green dots represent the downregulated genes, while the red dots represent the upregulated genes. Heatmap of the expression of differentially expressed **D** mRNAs, **E** lncRNAs, and **F** circRNAs

In total, 15,454 mRNA, 12,286 lncRNA, and 10,813 circRNA reads were identified. The expression matrix was shown in Additional file 1: Table S1. Through setting the criteria |log_2_FC|> 1.0 and adjusted p-value (Q value) < 0.05 using DESeq2 package, we obtained 135 mRNAs (89 upregulated and 46 downregulated), 132 lncRNAs (115 upregulated and 17 downregulated), and 359 circRNAs (292 upregulated and 67 downregulated), which were differentially expressed in the exosomes from patients with COPD compared with the controls. Refined information of the differentially expressed exosomal mRNAs, lncRNAs and circRNAs were listed in Additional file 2: Tables S2–S4. The top three significantly upregulated mRNAs in the COPD cohort were *PIGZ*, *NCBP2L*, and *THTPA*, while the top three significantly downregulated mRNAs were *VWF*, *FAM90A1*, and *RFXAP*. The volcano plot showed the expression patterns of the mRNAs, lncRNAs, and circRNAs between the two groups (Fig. 2A–C). The heatmaps depicted the expression levels of differentially expressed mRNAs, lncRNAs, and circRNAs in each sample (Fig. 2D–F). Pearson’s correlation test was applied to the differentially expressed exosomal mRNAs and the clinical characteristics of the patients. As shown in the Additional file 3: Fig. S1, sputum cell counts, which reflect the lung microenvironment, were linearly associated with the number of differentially expressed mRNAs. Interestingly, upregulation of the same gene set, including *CD151*, *SPATA1*, *DEFA4*, *SERPINE3*, *FBXO3*, and *SART3*, was linearly associated with elevated counts of macrophages and lymphocytes and conversely decreased number of neutrophils in sputum from COPD patients. Meanwhile, the expressions of *AMN1*, *AP1AR*, and *CARMIL1* showed a linear correlation with the COPD Assessment Test (CAT) scores. In addition, *LSM5*, *ARID5A* were linearly positively correlated with counts of lymphocytes while linearly negatively correlated with counts of neutrophils in sputum. *CTNNA2* expression also showed linear positive correlation with numbers of eosinophils in sputum in COPD patients.

### Differentially expressed mRNAs are functionally enriched in immune-related pathways

**Fig. 3:**
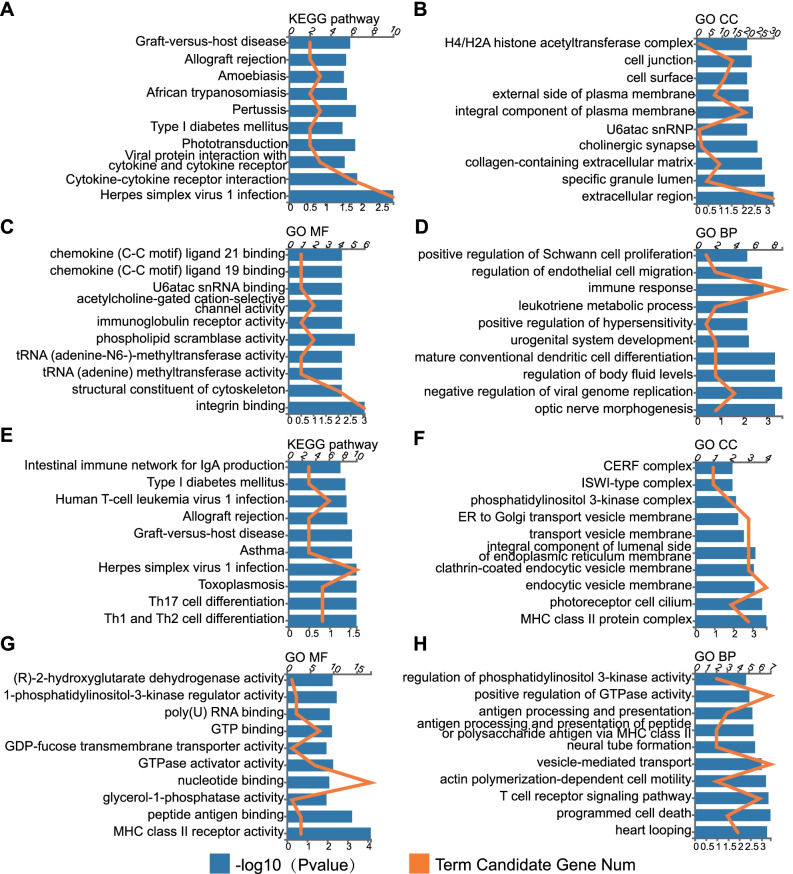
Functional analysis of differentially expressed mRNAs and target mRNAs of differentially expressed lncRNAs. The top 10 KEGG terms (**A**), GO-CC terms (**B**), GO-MF terms (**C**), and GO-BP terms (**D**) of differentially expressed mRNAs. The top 10 KEGG terms (**E**), GO-CC terms (**F**), GO-MF terms (**G**), and GO-BP terms (**H**) of target mRNAs of differentially expressed lncRNAs. *KEGG* Kyoto Encyclopedia of Genes and Genomes, *GO* Gene ontology, *CC* cellular component, *MF* molecular function, *BP* biological process

GO functional and KEGG pathway enrichment analyses were performed to annotate the differentially expressed mRNAs. KEGG enrichment analyses of mRNA profiles identified *Herpes simplex* virus 1 infection as the most enriched pathway, followed by cytokine-cytokine receptor interaction, viral protein interaction with cytokine and cytokine receptor and Pertussis (Fig. 3A). The enriched GO terms were sorted into three domains: biological process (BP), molecular function (MF), and cellular component (CC). In the cellular component, the predicted targets were mainly associated with the extracellular region, the specific granule lumen, and the collagen-containing extracellular matrix (Fig. 3B). In the molecular function category, DEGs were primarily related to integrin binding (Fig. 3C). Furthermore, the immune response represented the maximum number of DEGs in the biological processes (Fig. 3D).

LncRNAs can function as antisense RNAs by forming complementary hybrids with target mRNAs to regulate their expression. Therefore, we predicted the potential target mRNAs of the lncRNAs through the program “rnaplex,” and 111 mRNAs were found. Subsequently, we performed GO and KEGG analyses on these 111 mRNAs to explore the potential functions of the dysregulated lncRNAs. KEGG enrichment analyses showed various pathways, including Th1 and Th2 cell differentiation and Th17 cell differentiation (Fig. 3E). GO analyses identified major histocompatibility complex (MHC) class II molecules as the most enriched pathways in terms of both CC and MF (Fig. 3F, G), and programmed cell death as the top BP (Fig. 3H). These results implied that the specific gene expression patterns of plasma-derived exosomes may participate in the regulation of peripheral immune activity in patients with COPD.

### CeRNA prediction and construction of a ceRNA network

**Fig. 4:**
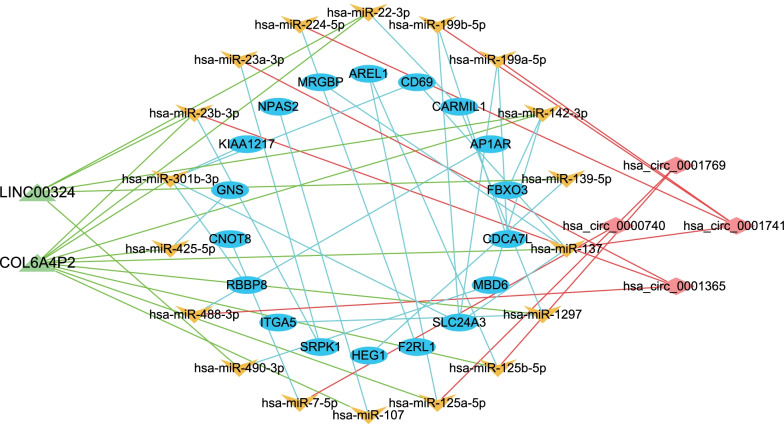
The lncRNA/circRNA–miRNA–mRNA ceRNA regulatory networks. Triangular nodes represent lncRNAs, V-shaped nodes represent miRNAs, elliptical nodes represent mRNAs, and rhombic nodes represent circRNAs. *miRNA* microRNA, *ceRNA* competing endogenous RNA

We obtained 282 miRNA-mRNA pairs, 153 miRNA-lncRNA pairs, and 68 miRNA-circRNA pairs for differentially expressed exosomal mRNAs, lncRNAs and circRNAs respectively by searching bioinformatic databases mentioned in methods. All of these RNA pairs were demonstrated in Additional file 4: Tables S5–S7. 26 miRNA-mRNA pairs having shared miRNA targets with miRNA-circRNA pairs or miRNA-lncRNA pairs were screened, and resultant 52 RNA pairs were put into Cytoscape software to construct a ceRNA network. Eventually, a ceRNA network consisting of 42 nodes which were 18 miRNAs, 18 mRNAs, two lncRNAs, and four circRNAs was built (Fig. 4).

### ScRNA-seq identified 18 distinct cell clusters from PBMCs

**Fig. 5:**
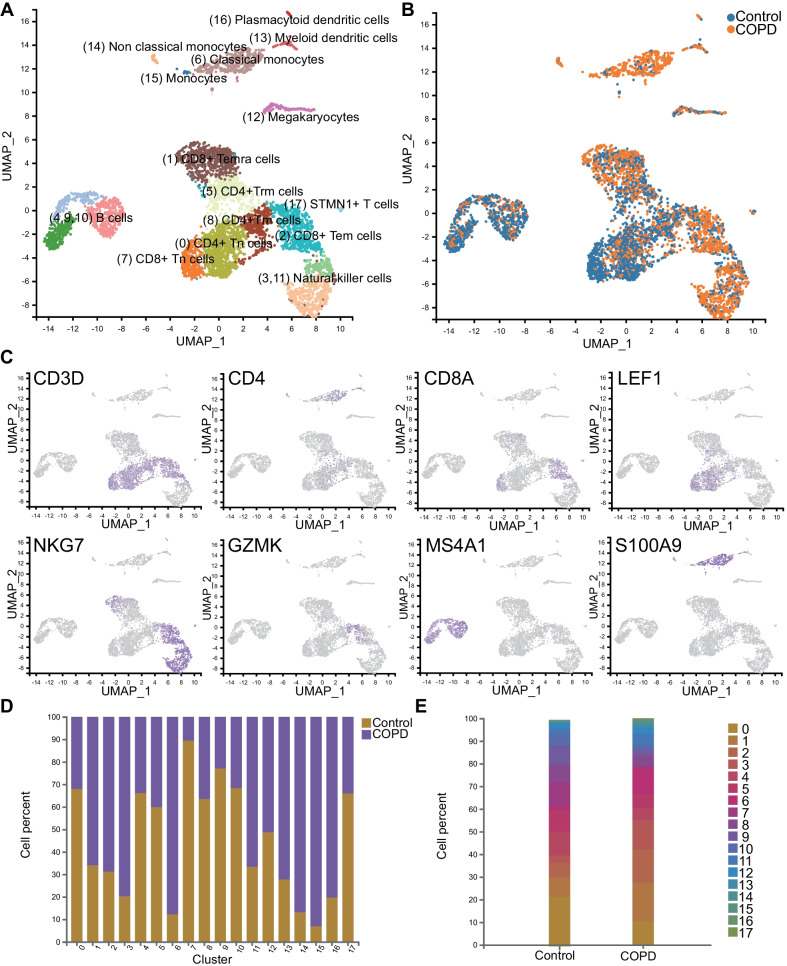
Single-cell unsupervised clustering and annotation. Uniform manifold approximation and projection (UMAP) representation of the pooled cells depicted in different colours by **A** cluster and cell type and **B** sample. **C** Canonical cell markers were used to annotate clusters. Each colour represents the expression level (grey means low and purple means high). **D** Bar plot of cell fractions in patients with COPD and healthy controls in each cluster. **E** Cell fractions of all clusters in patients with COPD and healthy controls

Next, we conducted single-cell transcriptomic analysis of PBMCs isolated from the patient Dis6 in the COPD group and Con4 in the control group, obtaining 8253 cells and 8349 cells, respectively. All samples were pooled and analysed through dimensional reduction using the graph-based visualisation method UMAP. Unsupervised cluster analysis of all cells identified 18 clusters in total, and all clusters were annotated predominantly based on the expression of known marker genes, as shown in Fig. 5A (resolution = 0.5). Cells from patients with COPD and healthy controls were also presented in different colours, as shown in Fig. 5B. These 18 clusters included three types of CD4 + T cells (clusters 0, 5, and 8), three types of CD8 + T cells (clusters 1, 2, and 7), two types of natural killer (NK) cells (clusters 3 and 11), three B cell populations(clusters 4, 9, and 10), three types of monocytes (clusters 6, 14, and 15), megakaryocytes (cluster 12), two subclusters of dendritic cells (clusters 13 and 16), and an ambiguous cluster characterised by a T cell marker (CD3D) without other representative marker genes (since it contained only 47 cells), which should have minor effects on further analyses.

Marker genes of each cluster are listed in the Table 2, and the expression levels of several representative markers are depicted in Fig. 5C. Among T cells, cluster 0 (CD4 +) and cluster 7 (CD8B +) showed CCR7 and LEF1 expression, which were considered markers of naïve T cells; cluster 1 exhibiting significant PRF1, KLRG1, and GZMH expression was annotated as CD8 + T effector memory CD45RA + (Temra) cells; cluster 2 (GZMK +) was defined as CD8 + effector memory T (Tem); cluster 5 (NR4A2 + , IL7R + , PTGER4 +) was annotated as CD4 + Trm cells; and cluster 8 (S100A4 + , LTB +) was recognised as CD4 + Tm cells [17, 18]. NK cells, including clusters 3 and 11, showed similar expression patterns and were located adjacently in UAMP. Clusters 4, 9, and 10 were annotated as B cells, marked by IGKC, IGHD, and MS4A1, respectively [19, 20]. Cluster 6 was defined as classical monocytes due to its high expression of LYZ, S100A9, and S100A8, while cluster 14 (FCGR3A +) was defined as non-classical monocytes [21, 22]. Cluster 12, marked by PPBP and PF4, was comprised of megakaryocytes [20]. Both groups of dendritic cells accounted for less than 1% of the cells; cluster 13 (HLA-DRB1 + , HLA-DRB5 +) was defined as myeloid dendritic cells, and cluster 16 (IRF7 + , IRF8 +) was annotated as plasmacytoid dendritic cells [21, 22].

**Table 2:** Identified clusters, annotated cell type, representative genes and their corresponding proportions

Cluster	Cell type	Representative genes	Proportion
0	CD4 + Tn	CCR7, LEF1	16.01%
5	CD4 + Trm	NR4A2, IL7R, PTGER4	7.79%
8	CD4 + Tm	S100A4, LTB	6.17%
1	CD8 + Temra	PRF1, KLRG1, GZMH	12.91%
2	CD8 + Tem	GZMK	10.53%
7	CD8 + Tn	CCR7, LEF1	6.25%
3	Natural killer cell	GNLY, NKG7	8.04%
11	Natural killer cell	GNLY, NKG7	3.52%
4	B cell	IGKC	7.94%
9	B cell	IGHD	5.52%
10	B cell	MS4A1	3.73%
6	Classical Monocyte	CD14, S100A9, LYZ	6.30%
14	Nonclassical Monocyte	CD16, FCGR3A, TCF7L2	0.64%
15	Monocyte	CD14, CLEC12A	0.43%
12	Megakaryocyte	PPBP, PF4	2.64%
13	Myeloid dendritic cell	HLA-DRB1, HLA-DRB5	0.87%
16	Plasmacytoid dendritic cell	IRF7, IRF8	0.43%
17	STMN + T cell	CD3D, STMN	0.28%

*Tn* Naïve T cell, *Trm* resident memory T cell, *Tm* memory T cell, *Temra* effector memory RA + T cell, *Tem* effector memory T cell

### Divergent cell composition between patients with COPD and healthy controls

The corresponding proportions of cells for each cluster are shown in Fig. 5D, E. Surprisingly, monocytes (clusters 6, 14, and 15) and dendritic cells (clusters 13 and 16) predominantly originated from patients with COPD. NK cells (clusters 3 and 11) also comprised more cells from patients with COPD. Conversely, B cells (clusters 4, 9, and 10) and CD4 + T cells (clusters 0, 5, and 8) mainly belonged to healthy controls. Notably, in terms of CD8 + T cells, cluster 1 (Temra) and cluster 2 (Tem) had a higher proportion of cells from patients with COPD, whereas cluster 7 (naïve CD8 + T cells) was almost exclusively populated with cells from the normal controls.

**Fig. 6:**
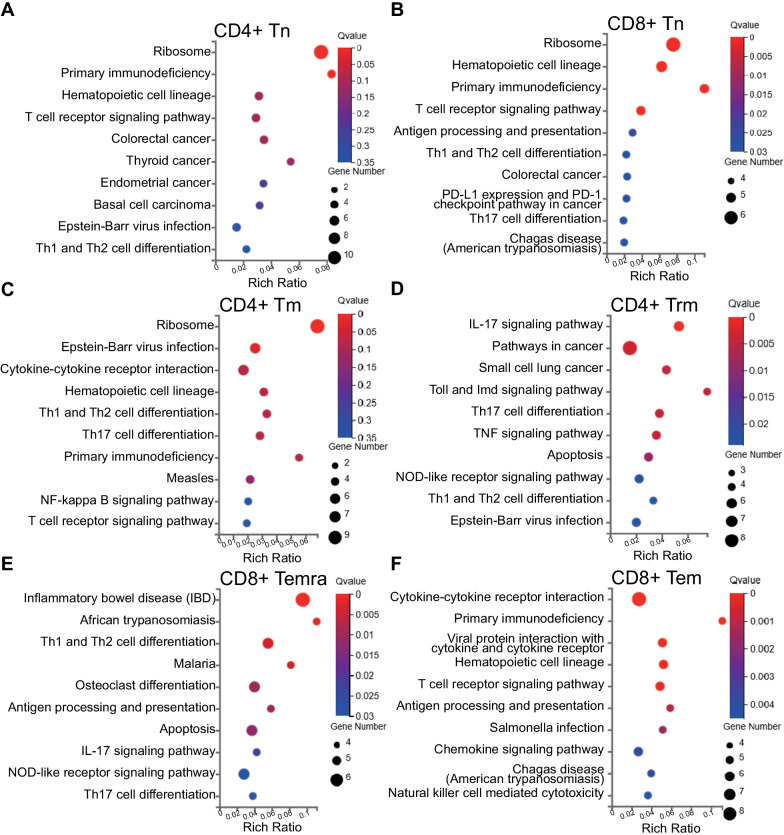
Functional analysis of marker genes in each T cell cluster. Bubble plot of KEGG showing the biological function of **A** CD4 + Tn, **B** CD8 + Tn, **C** CD4 + Tm, **D** CD4 + Trm, **E** CD8 + Temra, and **F** CD8 + Tem. The dot sizes represent enrichment abundance while the colours represent the Q values (blue to red). *KEGG* Kyoto Encyclopedia of Genes and Genomes, *Tn* Naïve T cell, *Tm* Memory T cell, *Trm* Resident memory T cell, *Temra* Effector memory RA + T cell, *Tem* effector memory T cell

Three types of CD8 + T cells (29.69%), three CD4 + T cell populations (29.97%), three B cell subtypes (17.19%), and two types of NK cells (11.56%) accounted for the majority of the identified cells, which may indicate their roles in COPD development and progression. The results of the KEGG functional analysis of the markers of these clusters are shown in Fig. 6, and the rest are shown in Additional file 5: Fig. S2. Cluster 0 (naive CD4 + T cells), cluster 7 (naive CD8 + T cells), and cluster 8 (CD4 + Tm) were mostly enriched in the ribosome pathway, whereas cluster 5 (CD4 + Trm) was involved in the IL-17 signalling pathway (Fig. 6A–D). In contrast, cluster 1 (CD8 + Temra) and cluster 2 (CD8 + Tem), whose proportions were higher in patients with COPD, were related to inflammation-related pathways, including inflammatory bowel disease (IBD) and cytokine-cytokine receptor interaction (Fig. 6E, F). Three clusters of B cells were enriched in the same terms, including haematopoietic cell lineage, asthma, and intestinal immune network for IgA production. NK cells (clusters 3 and 11) were enriched in antigen processing, presentation, and cytotoxic functions. Monocytes (clusters 6, 14, and 15) were comprised of phagosomes. Cluster 17, marked by STMN, was significantly related to the cell cycle, while cluster 12 was chiefly associated with platelet activation. Clusters 13 and 16, representing dendric cells, were mostly related to asthma, which may indicate some common pathways between COPD and asthma.

### Differential functional annotation of specific cell types between patients with COPD and healthy controls

**Fig. 7:**
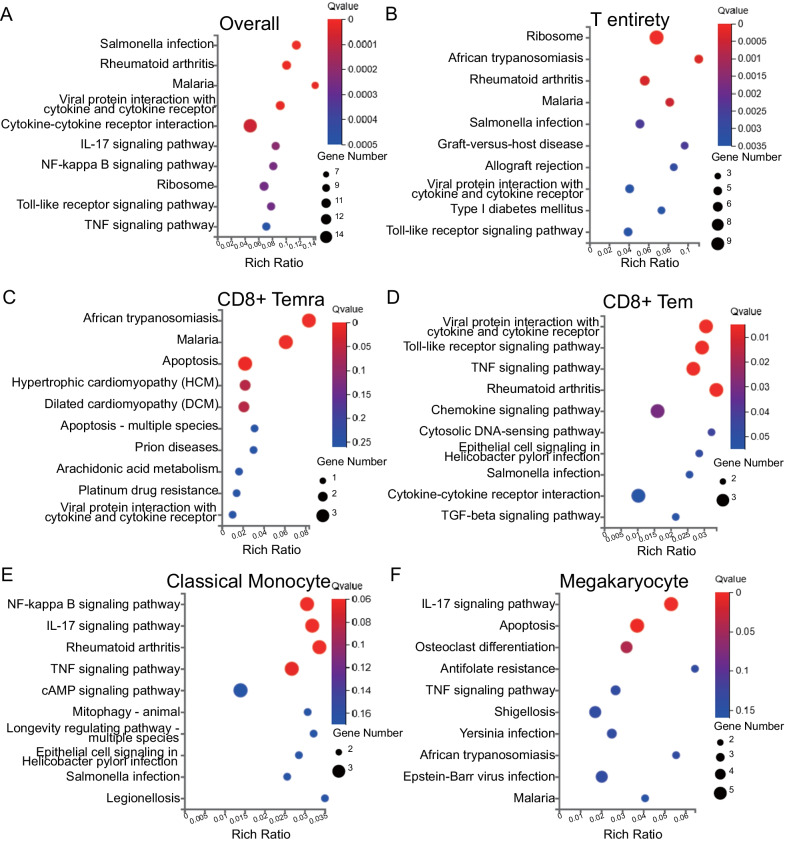
Functional analysis of DEGs in certain cell clusters between patients with COPD and healthy controls. Bubble plot of KEGG showing the biological function of DEGs in **A** all cells, **B** T cells, **C** CD8 + Temra, **D** CD8 + Tem, **E** classical monocytes, and **F** megakaryocytes. The dot sizes represent enrichment abundance while the colours represent the q values (blue to red). *DEGs* differentially expressed genes, *KEGG* Kyoto Encyclopedia of Genes and Genomes, *Temra* effector memory RA + T cell, *Tem* effector memory T cell

We identified 140 DEGs between patients with COPD and healthy controls, of which 104 were upregulated and 36 were downregulated in COPD (Additional file 6: Table S8). The top five upregulated genes were *LYZ*, *S100A9*, *CXCL8*, *S100A8*, and *PTGS2*, while the top five downregulated genes were *HBB*, *IGKC*, *IGLC3*, *IGLC2*, and *IGHM*. We then performed KEGG pathway analysis for DEGs at different levels, from individual cluster to pooled cell types. Overall analyses of all cells revealed that the DEGs were mainly enriched in *Salmonella* infection, rheumatoid arthritis, viral protein interaction with cytokines and cytokine receptors, malaria, and cytokine-cytokine receptor interaction (Fig. 7A).

Considering that T cells accounted for over half of the total cells and possessed the most divergent populations, we performed a detailed functional analysis of the DEGs in each T cell cluster defined by different functional states. Among all T cells in patients with COPD verse healthy controls, *KLRD1*, *GZMB*, *NKG7*, *GNLY*, and *CCL5* were the top five upregulated genes, whereas *HBB*, *HBA2*, *LTB*, *RPS13*, and *HBA1* were the top five downregulated genes. Pathways including ribosomes, African trypanosomiasis, malaria, *Salmonella* infection, and rheumatoid arthritis, a type of autoimmune disease, were significantly enriched in T cell entirety from patients with COPD (Fig. 7B). For a particular cell cluster, cluster 1 (CD8 + Temra) was enriched in apoptosis, whereas cluster 2 (CD8 + Tem) was associated with viral infections, Toll-like receptor signalling, and TNF signalling (Fig. 7C, D). DEGs from other clusters of T cells tended to be enriched in less specific pathways, as shown in the Additional file 7: Fig. S3. Compared with healthy controls, NK cells from patients with COPD may be more involved in cytokine-related interactions and play important roles in communicating with T cells. We also found that monocytes, especially cluster 6 (classical monocytes) from patients with COPD, were enriched in NF-κB signalling pathway, TNF signalling pathway, and rheumatoid arthritis (Fig. 7E). As for megakaryocytes, the DEGs were mainly enriched in the IL-17 signalling pathway and apoptosis (Fig. 7F), which suggests that monocytes and platelets may play a role in the pathophysiology of COPD, as reported previously [6]. DEGs from dendritic cells were enriched in viral protein, Th1, Th2, and Th17 cell differentiation, and the T cell receptor signalling pathways, indicating their role in viral immunity and the activation of T cell differentiation. There were only four DEGs (|log_2_FC|≤ 0.4) in B cells, suggesting that B cells may not be as multifunctional as T cells in the development of COPD, which was congruent with the results of functional analyses that showed no significant difference among the three B cell populations.

### Construction of a protein–protein interaction network combining exosomal and sc-RNA transcriptomics and clinical characteristics

**Fig. 8:**
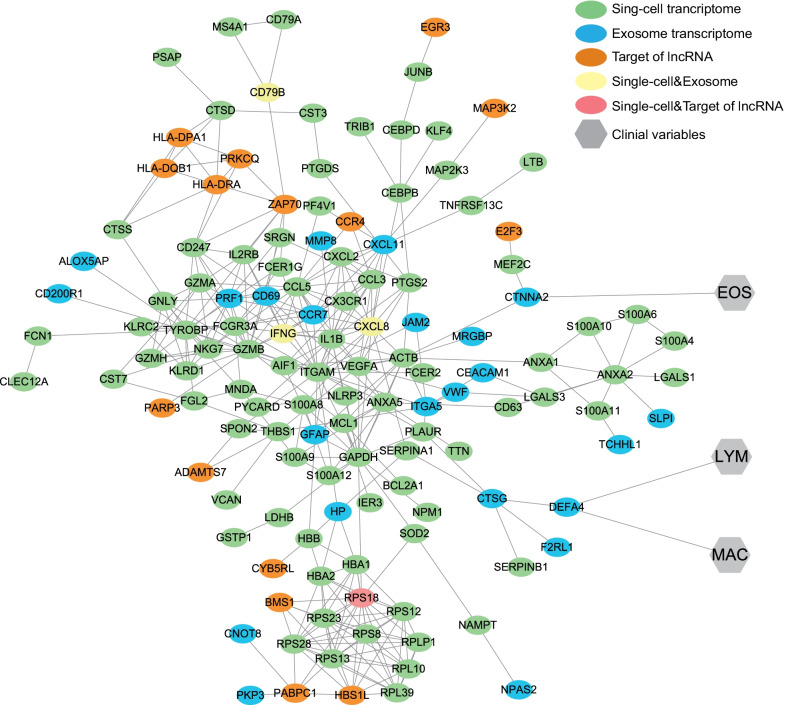
Protein–protein interaction network and its connection with sputum clinical variables. Elliptical nodes represent proteins, hexagonal nodes represent clinical variables, and the colours represent libraries. *EOS* eosinophil, *LYM* lymphocyte, *MAC* macrophage

To explore the connection between exosomal transcriptomics and sc-RNA transcriptomics, we constructed a protein–protein interaction (PPI) network by merging the DEGs of the two libraries and the target genes of the differentially expressed lncRNAs in exosomes using the Search Tool for the Retrieval of Interacting Genes (STRING) (https://string-db.org), with a minimum required interaction score of 0.700 (high confidence). All the genes included for PPI network construction were listed in Additional file 6: Table S9. The network was visualised using Cytoscape software. Eventually, a PPI network containing 130 nodes (genes) and 348 edges (interactions between genes) was constructed (Fig. 8). From this network, we observed a relatively tight interaction between the two transcriptomes. Reflecting on the exosomal mRNAs presenting significant correlation with clinical characteristics (Additional file 3: Fig. S1), *CTNNA2* and *DEFA4* also appeared in the PPI network and their clinical relevance were tagged. Among all the nodes, *CXCL8*, *IFNG*, and *CD79B* were genes common between the two DEG sets, whereas *RPS18* was shared by DEGs in the sc-RNA library and the target genes of the differentially expressed lncRNAs in exosomes.

## Discussion

Our study provides an in-depth RNA profile of exosomes in plasma and single-cell transcriptomic atlas of PBMCs in COPD, affording insights into the local and the peripheral immune status of COPD, which we hope may assist in ultimately elucidating the mechanisms underpinning the development of COPD. To the best of our knowledge, this is the first study to characterise PBMC transcriptome at single-cell resolution in COPD by using scRNA sequencing. Reasoning that exosome may serve as messengers of lung tissues, we also attempted to identify the connection between plasma exosomal RNA and PBMC transcriptome in COPD for the first time.

First, we profiled exosomal RNAs in COPD and identified several gene signatures associated with COPD clinical characteristics. Then, we used scRNA sequencing of PBMCs to depict the peripheral immune niche in COPD and constructed a PPI network to further explore the connection between exosomes and PBMCs. It could be implied from our results that exosomes might play an intermediate role in interactions between the systemic immune response and local lung tissues meanwhile the systemic immunity may mutually influence pulmonary immune microenvironment in COPD patients.

We discovered a gene array composed of *CD151*, *SPATA1*, *DEFA4*, *SERPINE3*, *FBXO3*, and *SART3*, which were related to higher macrophage, higher lymphocyte, and lower neutrophil cell counts in the sputum, indicating the possibility of airway leucocyte recruitment, which to some extent concurred with the pathways enriched by differentially expressed exosomal mRNAs concerning lymphocyte function such as allograft rejection, Viral protein and so on (Fig. 3A). *CD151* encodes a cell surface protein involved in integrin-mediated cell adhesion and promotes macrophage infiltration [23]. *DEFA4* belongs to the defensin family, which is abundant in the granules of neutrophils that defend the host against bacteria; nevertheless, its function has not been reported in COPD. It has been discovered that circulating FBXO3 could potentially stimulate cytokine release from inflammatory cells in patients with sepsis [24]. SART3 antigen was found to be expressed in human CD34 + cells, enabling the recruitment of cytotoxic T cells and triggering pro-inflammatory responses [25]. Expression of *AMN1*, *AP1AR*, and *CARMIL1* were associated with the CAT score, which reflects the severity of COPD and *CARMIL1* has been shown to be involved in inflammatory signalling, specifically IL-1 signal transduction [26]. The above results suggested the potential roles of exosomal mRNAs in promoting leucocyte migration and inflammatory signal transmission within the pulmonary tissue in patients with COPD, although most of the gene targets have not been explored in exosomes, which warranted massive experiments to verify.

KEGG and GO enrichment analyses showed that the differentially expressed exosomal mRNAs were enriched to a large extent in signal transduction and immune responses. For example, the top three KEGG enriched pathways were concentrated on viral infection and cytokines, either in the level 1 or level 2 categories. The effects of viral infection in COPD have been described before; viruses could curtail phagocytosis by macrophages and reduce cytokine production [27], thus making patients with COPD more vulnerable to bacterial infection. Programmed cell death in COPD has been characterised as another feature of COPD development, which is triggered by cigarette smoke, often followed by persistent inflammation, owing to the release of damage-associated molecular patterns (DAMPs) [28]. KEGG analysis showed enrichment in Th1, Th2, and Th17 cell differentiation, the proportions of which were reported to increase in patients with COPD [29]. Moreover, another study also showed that different inflammatory phenotypes may be related to different bacterial species in the airways of patients with COPD [30]. The ceRNA network illustrated the possible interactions in between exosomal RNAs. LINC00324 is capable of promoting lung cancer cell proliferation, counteracted by miR-615-5p (Fig. 4) [31]. All of the miRNA nodes have been reported to exert roles in immunity, tumorigenesis, or both [32–34]. Defense against infection relies on immunity, in the same context that cancer surveillance largely depends on immunity. Therefore, it could be implied that exosome contained RNAs may affect homeostasis maintenance through communication with each other and regulation of immune signaling pathways.

ScRNA sequencing of PBMCs in our study provided a panoramic albeit obscure view of systemic immune status in patients with COPD (Fig. 5). Many of the cell clusters we identified including cluster 14 (TCF7L2 + , CD16 + non-classical monocytes) and cluster 15 (CLEC12A + , CD14 + monocytes) have not been reported in COPD. The maturity of dendritic cell subsets (clusters 13 and 16, higher in COPD) indicated by the increased expression of co-stimulatory molecules was found to be positively correlated with COPD severity [35]. All of the refined clusters of NK cells and B cells have been described in COPD. Our study indicated that the number of NK cells (cluster 3,11) with more robust cytotoxic effects may increase in patients with COPD (Fig. 5D). Three subclusters of B cells all increased in COPD patient in our study, consistent with the former findings that B cells were higher in COPD pulmonary tissues [36]. However, B cells of different subclusters may play paradoxical roles in COPD. On the one hand, they may produce autoantibodies directed against lung cells; on the other hand, they could elicit adaptive immune responses against pathogens [36], which warrants further study.

In terms of T cell subtypes, our results were concordant with previous studies that patients with COPD have shrinking naïve T cell pools, regardless of CD4 or CD8 positivity and CD8 + Temra and CD8 + Tem markedly elevate in COPD patients [37]. In addition, we found the decline of peripheral CD4 + Trm (cluster 5) and CD4 + Tm (cluster 8, ANXA1 +) in COPD patient and evidence has shown that CD4 + Trm (cluster 5) could respond rapidly to pathogen re-exposure and facilitate CD8 + T cell recruitment [38], suggesting its importance in protecting the host against infection. The protein expression of ANXA1, which is considered as an anti-inflammatory factor, was found to be increased in the bronchoalveolar lavage (BAL) of patients with COPD [39]. Among all six T cell clusters, only cluster 1 (CD8 + Temra) and cluster 2 (CD8 + Tem) were more abundant in patients with COPD; the other clusters showed the opposite. According to enrichment analyses of cell markers, clusters 1 and 2 were related to inflammation pathways, whereas naïve T cells were enriched in the ribosome pathway, suggesting higher translation activity. Taken together, it may be rationale to assume that T cell subsets with either higher differentiation potential, such as naïve T cells, or with faster response to pathogens, such as CD4 + Trm may decline in COPD patients. Conversely, CD8 + Temra subset, representative of low proliferative and reactive capacity especially to virus[40], and Tem marked with advanced differentiation, may be predominant in COPD patients. Based on their developmental trajectory, we postulated that progressive depletion of naïve T cell pools in COPD maybe caused by repeated exposure to pathogens, resulting a shift from naïve T cells to Tem and Temra. Consequently, patients with COPD may have expanded Temra and Tem cell subsets, which could further exhaust their reserved capacity to respond to pathogens, which remains to be confirmed.

Considering PBMCs as a whole, the top five upregulated genes were *LYZ*, which encodes an antibiotic lysozyme, *S100A9*, *CXCL8*, *S100A8*, and *PTGS2*. *S100A8/9* play roles in many aspects of immunity, such as the induction of cytokine cascades, stimulation of leucocyte chemotaxis, and antimicrobial activity [41]. Surprisingly, functions of down-regulated genes in COPD were coincident in encoding immunoglobulins, indicating a possible declined capacity of PBMCs in generating antibodies. Furthermore, immunoglobulin level was reported to be elevated in local pulmonary tissue in COPD patients [42], which from another side indicate the overwhelmed immune response in COPD. In terms of T cells, the pathways enriched by cluster 2 (CD8 + Tem) DEGs seem to have robust interconnections (Fig. 7D). Viral infections could lead to the emission of DAMPs, which activate Toll-like receptors and other downstream effectors regulated by TNF receptor-associated factor (TRAF) [43]. Pathways enriched by DEGs of T cells across subclusters were predominantly associated with *Salmonella* infection and rheumatoid arthritis (Fig. 7B). It has been reported that the response to *Salmonella* species and other gram-negative bacteria is correlated with lung function parameters and cigarette smoking [44]. Moreover, COPD may encompass some features of autoimmune diseases like RA [45], since patients with COPD also have a higher risk for incident RA [46].

PPI network (Fig. 8), *CXCL8*, *IFNG*, *CD79B*, and *RPS18* merit attention, considering their dual identity as molecular signatures in both exosome and PBMC. All of these genes participate in immune reactions especially defending against viral or microbial infections. Based on previous evidence and our findings, we hypothesized that COPD development may be initially induced by exposure to pathogens and other individual factors, such as cigarette smoke, air pollution and immune-compromised airway, whose signals could be transduced from local lung tissue to peripheral immune system via exosomes and other mediators. Long-term pathogenic stimuli might dampen innate immunity, the first barrier against pathogens in COPD patients like phagocytosis by macrophages, resulting prolonged infection, resorting to adaptive immune system. As a result, continuous exposure to antigens could cause the depletion of peripheral naïve T cell pools in patients with COPD, depriving their capacity to elicit rapid and robust responses against pathogens, instead increasing their susceptibility to bacterial and viral pathogens, which forms a vicious circle.

Our research has several limitations. An obvious limitation is the small sample size, which only sequenced exosomes from six patients and profiled single-cell transcriptome in one patient. Our findings definitely need more studies with larger sample size for validation. Second, the isolated exosomes in our study were relatively sparse, which limited the amount of exosomal information available. Exosome as a new hotspot of medical research emerged in the last decade, is still lacking effective means of isolation, demanding more efforts to upscale this technology. Finally, our analyses were virtually based on comprehensive sequencing profiles, albeit without further validation, which warrants a large number of experimental verification in the future.

## Conclusions

By sequencing exosomal RNA and single-cell transcriptome of PBMC in COPD, we characterised several possible molecular signatures for COPD, delineated the potential interconnection between impaired immune function and COPD progression, and identified the possible role of exosomes in mediating local and systemic immune reactions, which requires extensive experimentation and larger-scale COPD population for validation.

## Supplementary Information

**Additional file 1: Table S1.** The expression matrix of exosomal RNA sequencing.

**Additional file 2: Table S2.** Exosomal differentially expressed mRNA between COPD patient and healthy control. **Table S3.** Exosomal differentially expressed lncRNA between COPD patient and healthy control. **Table S4.** Exosomal differentially expressed circRNA between COPD patient and healthy control.

**Additional file 3: Fig. S1.** Correlation between expression level of differentially expressed mRNA and clinical variables. The significant correlations between differentially expressed mRNA and clinical variables were plotted. CAT: COPD assessment test, sneu: sputum neutrophil, slym: sputum lymphocyte, smac: sputum macrophage, seos: sputum eosinophil.

**Additional file 4: Table S5.** Pairs of mRNA-miRNA predicted by TargetScan. **Table S6.** Pairs of lncRNA-miRNA predicted by miRcode. **Table S7.** Pairs of circRNA-miRNA predicted by starBase.

**Additional file 5: Fig. S2.** Functional analysis of marker genes in other clusters. Bubble plot of KEGG showing the biological function of (A) cluster 3, (B) cluster 4, (C) cluster 6, (D) cluster 9, (E) cluster 10, (F) cluster 11, (G) cluster 12, (H) cluster 13, (I) cluster 14, (J) cluster 15, (K) cluster 16, and (L) cluster 17. The dot sizes represent enrichment abundance while the colours represent the Q values (blue to red). KEGG: Kyoto Encyclopedia of Genes and Genomes.

**Additional file 6: Table S8.** Single-cell differentially expressed mRNA in overall cells between COPD patient and healthy control. **Table S9.** Gene list for construction of protein–protein interaction network

**Additional file 7: Fig. S3.** Functional analysis of DEGs in other clusters between patients with COPD and healthy controls. Bubble plot of KEGG showing the biological function of DEGs in (A) cluster 0, (B) cluster 3, (C) cluster 5, (D) cluster 7, (E) cluster 8, (F) cluster 11, (G) cluster 13, (H) cluster 16. The dot sizes represent enrichment abundance while the colours represent the Q values (blue to red). DEGs: Differentially expressed genes, KEGG: Kyoto Encyclopedia of Genes and Genomes.

## Data Availability

The datasets used and/or analyzed during the current study are available from the corresponding author on reasonable request.

## Abbreviations

COPD: Chronic obstructive pulmonary disease
PBMCs: Peripheral blood mononuclear cells
scRNA: Single-cell RNA
PBS: Phosphate-buffered saline
TEM: Transmission electron microscopy
nFCM: Nano-flow cytometry
ceRNA: Competing endogenous RNA
GEMs: Gel beads-in-emulsions
UMAP: Uniform manifold approximation and projection
GO: Gene Ontology
KEGG: Kyoto Encyclopedia of Genes
BP: Biological process
MF: Molecular function
CC: Cellular component
IBD: Inflammatory bowel disease
EMT: Epithelial-mesenchymal transformation
IGF1R: Insulin-like growth factor-1 receptor
BAL: Bronchoalveolar lavage
LPS: Lipopolysaccharides
TRAF: TNF receptor-associated factor
NK: Natural killer
PPI: Protein–protein interaction
Temra: Effector memory RA + T cell
Tem: Effector memory T cell
DEGs: Differentially expressed genes
