# Supplementary material for: Combining single-cell RNA sequencing of peripheral blood mononuclear cells and exosomal transcriptome to reveal the cellular and genetic profiles in COPD
Source: Respir Res. 2022 Sep 20;23:260. doi: 10.1186/s12931-022-02182-8 (PMC9490964; doi:10.1186/s12931-022-02182-8)
Supplement: Supplementary file 2 — Additional file 2: Table S2. Exosomal differentially expressed mRNA between COPD patient and healthy control. Table S3. Exosomal differentially expressed lncRNA between COPD patient and healthy control. Table S4. Exosomal differentially expressed circRNA between COPD patient and healthy control. [file 12931_2022_2182_MOESM2_ESM.docx]

Table S2. Exosomal differentially expressed mRNA between COPD patient and healthy control.

| Gene Symbol | Type | log2 (Disease / Normal) | Qvalue (Disease / Normal) |
| --- | --- | --- | --- |
| DPH3P1 | mRNA | -22.53 | 4.17E-11 |
| SPATA1 | mRNA | 22.89 | 6.03E-12 |
| IL18BP | mRNA | 11.42 | 5.11E-03 |
| YKT6 | mRNA | 10.55 | 1.20E-02 |
| ZNF274 | mRNA | 23.04 | 3.09E-11 |
| LOC107986004 | mRNA | 22.18 | 1.45E-10 |
| ARID5A | mRNA | 22.41 | 8.92E-12 |
| PKP3 | mRNA | 23.56 | 1.47E-11 |
| CMTM7 | mRNA | 22.58 | 6.46E-11 |
| CHRNB2 | mRNA | -10.80 | 2.73E-03 |
| MBD6 | mRNA | -22.73 | 3.03E-11 |
| CGAS | mRNA | 21.41 | 6.37E-10 |
| CKMT2 | mRNA | 9.32 | 3.26E-02 |
| CCR7 | mRNA | -22.73 | 3.03E-11 |
| SNX20 | mRNA | 22.81 | 2.47E-11 |
| TCHHL1 | mRNA | -23.27 | 1.37E-11 |
| PODN | mRNA | 10.47 | 2.41E-02 |
| FLACC1 | mRNA | -23.37 | 1.18E-11 |
| CD200R1 | mRNA | 11.55 | 1.30E-02 |
| TMEM155 | mRNA | 21.18 | 9.66E-10 |
| C7orf31 | mRNA | 22.92 | 3.64E-11 |
| TLDC2 | mRNA | 23.14 | 2.73E-11 |
| KBTBD3 | mRNA | 22.98 | 3.34E-11 |
| GCOM1 | mRNA | 22.97 | 3.37E-11 |
| PRSS36 | mRNA | 21.08 | 1.16E-09 |
| B3GALNT2 | mRNA | 10.67 | 6.43E-03 |
| PM20D1 | mRNA | 21.49 | 5.51E-10 |
| CTNNA2 | mRNA | -8.63 | 2.07E-02 |
| CTSG | mRNA | -22.51 | 4.35E-11 |
| FAM81B | mRNA | -23.07 | 1.86E-11 |
| CLECL1 | mRNA | 22.40 | 9.34E-11 |
| ZNF836 | mRNA | 20.93 | 1.40E-09 |
| DEFA4 | mRNA | 22.38 | 1.12E-11 |
| AMN1 | mRNA | 9.94 | 1.10E-02 |
| EPB42 | mRNA | -23.24 | 1.37E-11 |
| EPHB2 | mRNA | 22.82 | 4.32E-11 |
| F2RL1 | mRNA | -22.58 | 3.77E-11 |
| PPP1R35 | mRNA | 21.49 | 5.68E-10 |
| SIPA1L3 | mRNA | 9.64 | 3.34E-02 |
| MAN2B2 | mRNA | -23.28 | 1.36E-11 |
| LSM5 | mRNA | 22.24 | 1.03E-11 |
| ALOX5AP | mRNA | 10.74 | 1.30E-02 |
| ABI3BP | mRNA | -9.53 | 4.53E-02 |
| FBXO3 | mRNA | 10.74 | 1.16E-02 |
| ITGB1BP2 | mRNA | -22.76 | 2.90E-11 |
| GFAP | mRNA | -23.27 | 1.37E-11 |
| FOXB1 | mRNA | -8.81 | 4.82E-02 |
| GNS | mRNA | -8.81 | 1.33E-02 |
| CSNK2A3 | mRNA | -23.49 | 9.97E-12 |
| C12orf40 | mRNA | -22.47 | 4.59E-11 |
| OSTM1 | mRNA | -7.93 | 9.57E-03 |
| C11orf21 | mRNA | -22.73 | 3.03E-11 |
| HP | mRNA | -22.84 | 2.62E-11 |
| ANO9 | mRNA | 22.58 | 8.63E-12 |
| RFPL4A | mRNA | 21.65 | 3.91E-10 |
| IFNG | mRNA | 20.70 | 2.39E-09 |
| CXCL8 | mRNA | -6.92 | 2.02E-02 |
| ITGA5 | mRNA | -9.49 | 4.93E-02 |
| KCNA7 | mRNA | 20.47 | 4.08E-09 |
| ANKRD34C | mRNA | 21.08 | 1.15E-09 |
| NCBP2L | mRNA | 25.38 | 1.05E-12 |
| FSIP2 | mRNA | -9.75 | 3.88E-02 |
| LPO | mRNA | 23.01 | 3.21E-11 |
| PRR26 | mRNA | 10.63 | 3.41E-03 |
| SMCP | mRNA | 23.96 | 8.66E-12 |
| MGP | mRNA | 10.08 | 2.11E-02 |
| MMP8 | mRNA | 7.33 | 2.07E-02 |
| MARCHF11 | mRNA | 23.82 | 1.02E-11 |
| NPAS2 | mRNA | -22.54 | 4.12E-11 |
| OAS1 | mRNA | 23.28 | 2.20E-11 |
| OGN | mRNA | -23.37 | 1.62E-12 |
| TAS2R8 | mRNA | 23.58 | 1.41E-11 |
| IMPG2 | mRNA | 10.59 | 4.19E-02 |
| CLDN18 | mRNA | 21.72 | 3.67E-10 |
| PDE6G | mRNA | -22.63 | 3.47E-11 |
| TRMO | mRNA | 22.87 | 4.00E-11 |
| PLSCR1 | mRNA | -23.34 | 1.24E-11 |
| MRPL39 | mRNA | 9.14 | 2.12E-02 |
| LZTFL1 | mRNA | 23.98 | 8.63E-12 |
| ROPN1 | mRNA | 21.28 | 7.85E-10 |
| FAM90A1 | mRNA | -24.03 | 4.48E-12 |
| MRGBP | mRNA | 22.95 | 7.04E-12 |
| AP1AR | mRNA | 9.88 | 3.34E-02 |
| PRF1 | mRNA | -22.53 | 4.17E-11 |
| CDCA7L | mRNA | 9.79 | 1.81E-02 |
| CARMIL1 | mRNA | 9.70 | 1.90E-02 |
| HHAT | mRNA | -23.44 | 1.02E-11 |
| KIAA1217 | mRNA | -7.99 | 2.20E-02 |
| CELF4 | mRNA | 11.36 | 1.62E-02 |
| CHRNA10 | mRNA | -22.38 | 5.38E-11 |
| RHOJ | mRNA | -23.48 | 1.00E-11 |
| SLC24A3 | mRNA | 22.22 | 1.31E-10 |
| HEG1 | mRNA | 9.90 | 1.51E-02 |
| MRTFB | mRNA | 10.16 | 2.94E-02 |
| ELAPOR1 | mRNA | -9.01 | 2.50E-02 |
| CCDC191 | mRNA | 10.23 | 3.34E-02 |
| ADGRB2 | mRNA | -22.84 | 2.63E-11 |
| JAM2 | mRNA | -23.11 | 2.34E-12 |
| RBBP8 | mRNA | 10.54 | 7.85E-04 |
| RFXAP | mRNA | -23.75 | 7.17E-12 |
| CEACAM1 | mRNA | 10.88 | 2.20E-02 |
| CXCL11 | mRNA | 22.22 | 5.18E-11 |
| ZBED8 | mRNA | 22.03 | 1.93E-10 |
| DPEP2 | mRNA | 22.12 | 1.61E-10 |
| SGTA | mRNA | 10.48 | 4.35E-02 |
| SERPINE3 | mRNA | 20.91 | 1.21E-10 |
| SLC6A1 | mRNA | 22.29 | 1.16E-10 |
| SCGB1C2 | mRNA | -10.38 | 1.49E-02 |
| SLPI | mRNA | 22.81 | 4.48E-12 |
| DDRGK1 | mRNA | 8.02 | 1.40E-02 |
| SOX15 | mRNA | 21.50 | 5.53E-10 |
| SPTB | mRNA | -22.74 | 3.60E-12 |
| SRPK1 | mRNA | 7.41 | 2.27E-02 |
| LOC728392 | mRNA | 23.18 | 2.62E-11 |
| VWF | mRNA | -24.11 | 7.34E-13 |
| ZNF185 | mRNA | -23.59 | 8.66E-12 |
| ZNF200 | mRNA | 10.95 | 7.58E-03 |
| THTPA | mRNA | 23.99 | 5.84E-13 |
| ZNF768 | mRNA | -9.09 | 3.60E-02 |
| PIGZ | mRNA | 25.54 | 8.00E-13 |
| EFHC2 | mRNA | 21.30 | 7.85E-10 |
| ZNF514 | mRNA | 21.05 | 1.21E-09 |
| TNFRSF25 | mRNA | 21.43 | 6.22E-10 |
| HCAR3 | mRNA | -7.90 | 1.15E-02 |
| ZNF799 | mRNA | 23.09 | 2.90E-11 |
| SEC11C | mRNA | 22.23 | 1.28E-10 |
| CALML4 | mRNA | 21.04 | 1.25E-09 |
| CNOT8 | mRNA | 6.78 | 1.97E-02 |
| TNFSF8 | mRNA | 22.52 | 7.11E-11 |
| MED20 | mRNA | -22.46 | 4.65E-11 |
| CD69 | mRNA | 22.29 | 1.16E-10 |
| SART3 | mRNA | 7.31 | 3.89E-02 |
| CD79B | mRNA | 21.53 | 5.22E-10 |
| CD151 | mRNA | 23.06 | 8.24E-12 |
| AREL1 | mRNA | 6.99 | 2.90E-02 |

Table S3. Exosomal differentially expressed lncRNA between COPD patient and healthy control.

| Gene Symbol | Type | log2 (Disease / Normal) | Qvalue (Disease / Normal) |
| --- | --- | --- | --- |
| LINC01176 | lncRNA | 22.93 | 3.58E-11 |
| ZSWIM8-AS1 | lncRNA | 23.13 | 2.75E-11 |
| LOC101927237 | lncRNA | 21.47 | 5.83E-10 |
| LINC01781 | lncRNA | 24.53 | 3.74E-12 |
| RNF139-AS1 | lncRNA | 21.29 | 7.96E-10 |
| LINC01637 | lncRNA | 21.65 | 4.13E-10 |
| CTD-2194D22.4 | lncRNA | 21.67 | 3.74E-10 |
| LOC101929770 | lncRNA | 11.28 | 1.89E-02 |
| LOC102723846 | lncRNA | 10.46 | 4.64E-02 |
| LOC102724625 | lncRNA | -22.76 | 2.93E-11 |
| LOC105369302 | lncRNA | 21.26 | 8.27E-10 |
| LOC105369325 | lncRNA | 23.93 | 4.42E-12 |
| LOC105369842 | lncRNA | 23.62 | 1.37E-11 |
| LINC00385 | lncRNA | 21.11 | 1.09E-09 |
| LOC105370322 | lncRNA | 21.17 | 9.80E-10 |
| LOC105370460 | lncRNA | 22.06 | 1.84E-10 |
| LOC105370512 | lncRNA | 21.37 | 6.94E-10 |
| LOC105371068 | lncRNA | -23.16 | 1.56E-11 |
| LOC105371751 | lncRNA | -11.16 | 4.69E-03 |
| LOC105371773 | lncRNA | 23.61 | 1.37E-11 |
| LOC105371874 | lncRNA | 22.16 | 1.48E-10 |
| LOC105372185 | lncRNA | 22.90 | 3.74E-11 |
| LOC105373146 | lncRNA | 22.05 | 1.84E-10 |
| LOC105373255 | lncRNA | 21.36 | 7.09E-10 |
| LOC105374051 | lncRNA | 11.80 | 9.45E-03 |
| LOC105377596 | lncRNA | 21.19 | 9.50E-10 |
| LOC105377663 | lncRNA | 20.47 | 4.08E-09 |
| LOC105377743 | lncRNA | 21.94 | 2.30E-10 |
| LOC105378541 | lncRNA | 24.43 | 2.15E-12 |
| LOC105378805 | lncRNA | 22.35 | 1.03E-10 |
| LOC105379013 | lncRNA | 22.84 | 4.17E-11 |
| LOC105379085 | lncRNA | 21.17 | 9.62E-10 |
| LOC105379222 | lncRNA | -23.11 | 1.73E-11 |
| LOC105379880 | lncRNA | 21.47 | 5.87E-10 |
| MIR4432HG | lncRNA | 9.29 | 2.27E-02 |
| LOC107984120 | lncRNA | 25.12 | 1.62E-12 |
| LOC107984247 | lncRNA | -10.31 | 1.57E-02 |
| LOC107984251 | lncRNA | 21.26 | 8.30E-10 |
| LOC107984315 | lncRNA | 22.00 | 2.03E-10 |
| LOC107984428 | lncRNA | 22.50 | 7.53E-11 |
| LOC107984650 | lncRNA | 22.51 | 7.32E-11 |
| LOC107985037 | lncRNA | 21.35 | 7.30E-10 |
| LOC107985056 | lncRNA | 23.29 | 2.20E-11 |
| LOC107985508 | lncRNA | 22.55 | 6.91E-11 |
| LOC107985849 | lncRNA | 23.08 | 2.98E-11 |
| LOC107985986 | lncRNA | 23.35 | 2.01E-11 |
| LOC107986437 | lncRNA | 25.27 | 1.22E-12 |
| LOC107986924 | lncRNA | -25.09 | 5.84E-13 |
| LOC107987224 | lncRNA | 21.64 | 4.18E-10 |
| GSC-DT | lncRNA | 23.35 | 2.02E-11 |
| BMS1P4-AGAP5 | lncRNA | 21.45 | 6.12E-10 |
| CA5BP1-CA5B | lncRNA | 22.12 | 1.16E-10 |
| LINC00870 | lncRNA | 21.82 | 2.89E-10 |
| LINC00324 | lncRNA | 21.63 | 4.09E-10 |
| LINC00880 | lncRNA | 23.21 | 1.86E-11 |
| ADAMTS7P1 | lncRNA | 21.69 | 3.86E-10 |
| LINC01801 | lncRNA | 22.95 | 3.47E-11 |
| LINC01011 | lncRNA | -9.43 | 2.11E-02 |
| PRKCQ-AS1 | lncRNA | 20.75 | 2.15E-09 |
| SCAND2P | lncRNA | 21.19 | 9.50E-10 |
| LRRC37A4P | lncRNA | 9.71 | 4.60E-02 |
| LINC00862 | lncRNA | 21.12 | 1.09E-09 |
| FLJ32255 | lncRNA | -22.71 | 3.11E-11 |
| COL6A4P2 | lncRNA | -23.62 | 1.22E-12 |
| LINC00852 | lncRNA | -22.66 | 3.33E-11 |
| BGIG9606_100008 | lncRNA | 22.44 | 8.56E-11 |
| BGIG9606_100112 | lncRNA | 22.24 | 1.27E-10 |
| BGIG9606_100344 | lncRNA | 11.04 | 5.38E-03 |
| BGIG9606_100453 | lncRNA | 22.84 | 4.12E-11 |
| BGIG9606_101462 | lncRNA | -23.26 | 1.37E-11 |
| BGIG9606_101619 | lncRNA | 24.83 | 2.34E-12 |
| BGIG9606_102367 | lncRNA | 23.29 | 2.20E-11 |
| BGIG9606_103224 | lncRNA | 22.36 | 1.01E-10 |
| BGIG9606_103921 | lncRNA | -23.04 | 2.34E-12 |
| BGIG9606_104157 | lncRNA | 22.11 | 1.66E-10 |
| BGIG9606_104390 | lncRNA | 23.59 | 1.41E-11 |
| BGIG9606_104424 | lncRNA | 21.44 | 6.12E-10 |
| BGIG9606_68975 | lncRNA | -7.62 | 1.73E-02 |
| BGIG9606_69127 | lncRNA | 21.07 | 1.19E-09 |
| BGIG9606_69278 | lncRNA | 23.90 | 9.63E-12 |
| BGIG9606_71709 | lncRNA | 21.63 | 4.27E-10 |
| BGIG9606_72206 | lncRNA | 20.48 | 4.04E-09 |
| BGIG9606_72616 | lncRNA | 21.29 | 7.96E-10 |
| BGIG9606_73087 | lncRNA | 22.59 | 6.32E-11 |
| BGIG9606_73488 | lncRNA | 21.13 | 1.07E-09 |
| BGIG9606_74022 | lncRNA | 21.29 | 7.96E-10 |
| BGIG9606_74475 | lncRNA | 21.97 | 2.16E-10 |
| BGIG9606_74577 | lncRNA | 21.44 | 6.17E-10 |
| BGIG9606_75688 | lncRNA | 20.18 | 3.00E-10 |
| BGIG9606_75722 | lncRNA | 23.26 | 2.27E-11 |
| BGIG9606_77148 | lncRNA | 24.18 | 7.04E-12 |
| BGIG9606_77641 | lncRNA | 23.17 | 2.63E-11 |
| BGIG9606_78638 | lncRNA | 22.67 | 5.49E-11 |
| BGIG9606_78855 | lncRNA | 22.36 | 1.02E-10 |
| BGIG9606_79436 | lncRNA | 21.23 | 8.78E-10 |
| BGIG9606_80284 | lncRNA | 25.07 | 5.84E-13 |
| BGIG9606_81843 | lncRNA | 21.98 | 2.14E-10 |
| BGIG9606_82366 | lncRNA | 22.16 | 1.43E-10 |
| BGIG9606_82537 | lncRNA | 23.28 | 2.20E-11 |
| BGIG9606_83300 | lncRNA | 23.58 | 1.41E-11 |
| BGIG9606_83399 | lncRNA | 19.85 | 1.40E-08 |
| BGIG9606_83834 | lncRNA | 24.85 | 2.34E-12 |
| BGIG9606_84408 | lncRNA | 22.84 | 4.17E-11 |
| BGIG9606_84685 | lncRNA | -23.12 | 1.70E-11 |
| BGIG9606_85786 | lncRNA | 22.24 | 1.27E-10 |
| BGIG9606_86366 | lncRNA | 21.47 | 5.88E-10 |
| BGIG9606_87365 | lncRNA | 24.31 | 5.57E-12 |
| BGIG9606_87912 | lncRNA | 21.18 | 9.74E-10 |
| BGIG9606_88353 | lncRNA | 22.58 | 6.46E-11 |
| BGIG9606_88600 | lncRNA | 24.29 | 5.72E-12 |
| BGIG9606_89324 | lncRNA | 21.12 | 1.09E-09 |
| BGIG9606_90646 | lncRNA | 24.34 | 5.35E-12 |
| BGIG9606_90745 | lncRNA | 11.90 | 8.32E-03 |
| BGIG9606_91095 | lncRNA | 22.35 | 1.04E-10 |
| BGIG9606_91407 | lncRNA | 12.57 | 1.61E-03 |
| BGIG9606_91991 | lncRNA | 24.20 | 6.78E-12 |
| BGIG9606_92080 | lncRNA | 21.22 | 8.55E-10 |
| BGIG9606_92131 | lncRNA | 22.60 | 6.17E-11 |
| BGIG9606_92528 | lncRNA | 22.12 | 1.61E-10 |
| BGIG9606_92846 | lncRNA | 22.28 | 1.17E-10 |
| BGIG9606_93085 | lncRNA | 20.58 | 2.79E-09 |
| BGIG9606_93104 | lncRNA | 20.03 | 1.00E-08 |
| BGIG9606_93679 | lncRNA | 21.63 | 4.27E-10 |
| BGIG9606_93947 | lncRNA | 21.53 | 4.53E-10 |
| BGIG9606_94028 | lncRNA | 23.69 | 1.26E-11 |
| BGIG9606_94348 | lncRNA | 24.14 | 7.17E-12 |
| BGIG9606_95011 | lncRNA | 21.43 | 6.22E-10 |
| BGIG9606_95078 | lncRNA | 23.43 | 1.83E-11 |
| BGIG9606_96383 | lncRNA | -23.27 | 1.37E-11 |
| BGIG9606_97728 | lncRNA | 23.40 | 1.90E-11 |
| BGIG9606_98071 | lncRNA | -22.93 | 2.24E-11 |
| BGIG9606_99138 | lncRNA | -23.56 | 5.84E-13 |

Table S4. Exosomal differentially expressed circRNA between COPD patient and healthy control.

| Gene Symbol | Type | log2 (Disease / Normal) | Qvalue (Disease / Normal) |
| --- | --- | --- | --- |
| hsa_circ_0000238 | circRNA | 22.78 | 4.59E-11 |
| hsa_circ_0000415 | circRNA | -22.46 | 4.65E-11 |
| hsa_circ_0000466 | circRNA | 21.25 | 8.19E-10 |
| hsa_circ_0000553 | circRNA | -23.34 | 1.24E-11 |
| hsa_circ_0000640 | circRNA | 21.46 | 5.95E-10 |
| hsa_circ_0000740 | circRNA | -23.10 | 1.76E-11 |
| hsa_circ_0001069 | circRNA | 21.21 | 9.21E-10 |
| hsa_circ_0001333 | circRNA | -24.82 | 1.22E-12 |
| hsa_circ_0001365 | circRNA | 22.02 | 1.94E-10 |
| hsa_circ_0001741 | circRNA | 21.09 | 1.16E-09 |
| hsa_circ_0001769 | circRNA | 24.62 | 3.43E-12 |
| hsa_circ_0001785 | circRNA | 21.32 | 7.48E-10 |
| hsa_circ_0001789 | circRNA | 21.02 | 1.25E-09 |
| hsa_circ_0001885 | circRNA | 24.56 | 3.60E-12 |
| hsa_circ_0002028 | circRNA | 21.85 | 2.81E-10 |
| hsa_circ_0002330 | circRNA | 24.04 | 8.07E-12 |
| hsa_circ_0002365 | circRNA | 21.26 | 7.76E-10 |
| hsa_circ_0002443 | circRNA | 22.59 | 6.32E-11 |
| hsa_circ_0002517 | circRNA | 20.98 | 1.41E-09 |
| hsa_circ_0002536 | circRNA | 22.94 | 2.80E-11 |
| hsa_circ_0002622 | circRNA | -22.47 | 4.59E-11 |
| hsa_circ_0002646 | circRNA | 21.43 | 6.18E-10 |
| hsa_circ_0002676 | circRNA | 21.41 | 6.43E-10 |
| hsa_circ_0002881 | circRNA | 22.13 | 1.60E-10 |
| hsa_circ_0002910 | circRNA | 21.11 | 1.09E-09 |
| hsa_circ_0003056 | circRNA | 21.67 | 4.01E-10 |
| hsa_circ_0003183 | circRNA | 22.16 | 1.48E-10 |
| hsa_circ_0003194 | circRNA | 23.02 | 3.16E-11 |
| hsa_circ_0003381 | circRNA | 23.73 | 5.84E-13 |
| hsa_circ_0003388 | circRNA | 22.37 | 1.01E-10 |
| hsa_circ_0003400 | circRNA | 23.13 | 2.75E-11 |
| hsa_circ_0003669 | circRNA | 21.35 | 7.04E-10 |
| hsa_circ_0003891 | circRNA | 21.04 | 1.26E-09 |
| hsa_circ_0003950 | circRNA | 22.29 | 5.93E-11 |
| hsa_circ_0004098 | circRNA | 23.56 | 1.47E-11 |
| hsa_circ_0004197 | circRNA | 22.71 | 5.17E-11 |
| hsa_circ_0004349 | circRNA | 22.62 | 6.02E-11 |
| hsa_circ_0004391 | circRNA | 22.66 | 5.54E-11 |
| hsa_circ_0004966 | circRNA | -23.51 | 9.72E-12 |
| hsa_circ_0004979 | circRNA | -23.62 | 8.33E-12 |
| hsa_circ_0005136 | circRNA | 23.24 | 2.35E-11 |
| hsa_circ_0005154 | circRNA | 25.01 | 1.98E-12 |
| hsa_circ_0005505 | circRNA | -22.36 | 5.54E-11 |
| hsa_circ_0005573 | circRNA | 21.11 | 1.10E-09 |
| hsa_circ_0005615 | circRNA | -22.79 | 2.78E-11 |
| hsa_circ_0005718 | circRNA | 21.34 | 7.30E-10 |
| hsa_circ_0005765 | circRNA | 22.59 | 3.51E-11 |
| hsa_circ_0005769 | circRNA | 22.68 | 5.37E-11 |
| hsa_circ_0006096 | circRNA | 24.02 | 8.24E-12 |
| hsa_circ_0006097 | circRNA | -23.25 | 1.37E-11 |
| hsa_circ_0006396 | circRNA | 21.82 | 2.97E-10 |
| hsa_circ_0006512 | circRNA | 21.79 | 3.14E-10 |
| hsa_circ_0006599 | circRNA | 22.35 | 1.04E-10 |
| hsa_circ_0006670 | circRNA | 22.09 | 1.73E-10 |
| hsa_circ_0007044 | circRNA | 20.98 | 1.41E-09 |
| hsa_circ_0007348 | circRNA | 21.60 | 4.26E-10 |
| hsa_circ_0007581 | circRNA | 22.29 | 1.16E-10 |
| hsa_circ_0007912 | circRNA | 22.28 | 1.13E-10 |
| hsa_circ_0008141 | circRNA | -22.40 | 5.17E-11 |
| hsa_circ_0008351 | circRNA | 21.11 | 1.10E-09 |
| hsa_circ_0008371 | circRNA | 21.12 | 1.09E-09 |
| hsa_circ_0008696 | circRNA | 21.58 | 4.79E-10 |
| hsa_circ_0008701 | circRNA | -23.53 | 9.59E-12 |
| hsa_circ_0008772 | circRNA | 21.01 | 1.35E-09 |
| hsa_circ_0008827 | circRNA | 22.03 | 1.93E-10 |
| hsa_circ_0008881 | circRNA | -23.66 | 8.07E-12 |
| hsa_circ_0009020 | circRNA | 22.31 | 1.12E-10 |
| hsa_circ_0009168 | circRNA | -22.70 | 3.11E-11 |
| hsa_circ_0009699 | circRNA | -22.41 | 5.17E-11 |
| hsa_circ_0009700 | circRNA | -23.46 | 1.01E-11 |
| hsa_circ_0009770 | circRNA | 23.01 | 3.21E-11 |
| hsa_circ_0010403 | circRNA | 22.73 | 5.00E-11 |
| hsa_circ_0011117 | circRNA | -22.92 | 2.27E-11 |
| hsa_circ_0011752 | circRNA | 22.12 | 1.61E-10 |
| hsa_circ_0011879 | circRNA | 21.65 | 3.90E-10 |
| hsa_circ_0012134 | circRNA | 21.71 | 3.74E-10 |
| hsa_circ_0012135 | circRNA | 21.01 | 1.35E-09 |
| hsa_circ_0013470 | circRNA | 21.37 | 6.97E-10 |
| hsa_circ_0014369 | circRNA | 20.97 | 1.45E-09 |
| hsa_circ_0014401 | circRNA | 21.23 | 8.80E-10 |
| hsa_circ_0014402 | circRNA | 21.23 | 8.80E-10 |
| hsa_circ_0014490 | circRNA | 21.34 | 7.30E-10 |
| hsa_circ_0014491 | circRNA | 21.38 | 6.67E-10 |
| hsa_circ_0014633 | circRNA | 21.00 | 1.38E-09 |
| hsa_circ_0015022 | circRNA | -23.25 | 1.37E-11 |
| hsa_circ_0015466 | circRNA | -25.23 | 7.34E-13 |
| hsa_circ_0015662 | circRNA | -22.47 | 4.59E-11 |
| hsa_circ_0015733 | circRNA | 21.16 | 1.00E-09 |
| hsa_circ_0017329 | circRNA | -23.33 | 1.24E-11 |
| hsa_circ_0017850 | circRNA | 21.31 | 7.76E-10 |
| hsa_circ_0018542 | circRNA | 22.95 | 3.47E-11 |
| hsa_circ_0018634 | circRNA | -22.23 | 7.11E-11 |
| hsa_circ_0019520 | circRNA | 21.26 | 8.27E-10 |
| hsa_circ_0019521 | circRNA | 21.64 | 4.24E-10 |
| hsa_circ_0019522 | circRNA | 21.96 | 2.23E-10 |
| hsa_circ_0019785 | circRNA | 22.24 | 1.27E-10 |
| hsa_circ_0020727 | circRNA | 21.48 | 5.78E-10 |
| hsa_circ_0022201 | circRNA | 22.51 | 7.39E-11 |
| hsa_circ_0023694 | circRNA | -22.63 | 3.47E-11 |
| hsa_circ_0023881 | circRNA | 22.44 | 8.66E-11 |
| hsa_circ_0023886 | circRNA | 21.44 | 6.17E-10 |
| hsa_circ_0023892 | circRNA | 23.04 | 3.09E-11 |
| hsa_circ_0024256 | circRNA | 21.67 | 3.97E-10 |
| hsa_circ_0024812 | circRNA | 21.44 | 6.17E-10 |
| hsa_circ_0024948 | circRNA | 22.68 | 3.23E-11 |
| hsa_circ_0025311 | circRNA | 21.17 | 9.72E-10 |
| hsa_circ_0025312 | circRNA | 21.84 | 2.83E-10 |
| hsa_circ_0027353 | circRNA | 21.34 | 7.36E-10 |
| hsa_circ_0027525 | circRNA | -22.49 | 4.47E-11 |
| hsa_circ_0028058 | circRNA | 21.31 | 7.76E-10 |
| hsa_circ_0028282 | circRNA | 21.06 | 1.22E-09 |
| hsa_circ_0028587 | circRNA | 21.37 | 6.94E-10 |
| hsa_circ_0028935 | circRNA | 21.77 | 3.30E-10 |
| hsa_circ_0029855 | circRNA | 21.37 | 6.95E-10 |
| hsa_circ_0030049 | circRNA | 23.08 | 2.98E-11 |
| hsa_circ_0030121 | circRNA | 21.27 | 8.24E-10 |
| hsa_circ_0030286 | circRNA | 21.43 | 6.22E-10 |
| hsa_circ_0030287 | circRNA | 21.53 | 5.19E-10 |
| hsa_circ_0030799 | circRNA | -22.47 | 4.60E-11 |
| hsa_circ_0032840 | circRNA | 21.11 | 1.09E-09 |
| hsa_circ_0032992 | circRNA | -22.48 | 4.59E-11 |
| hsa_circ_0034745 | circRNA | -22.97 | 2.14E-11 |
| hsa_circ_0037367 | circRNA | 22.73 | 5.00E-11 |
| hsa_circ_0037368 | circRNA | 19.85 | 1.40E-08 |
| hsa_circ_0037943 | circRNA | 21.58 | 4.75E-10 |
| hsa_circ_0038045 | circRNA | 21.46 | 5.94E-10 |
| hsa_circ_0038803 | circRNA | 22.49 | 7.38E-11 |
| hsa_circ_0041598 | circRNA | 20.54 | 3.53E-09 |
| hsa_circ_0042255 | circRNA | 20.70 | 2.27E-09 |
| hsa_circ_0042702 | circRNA | -22.94 | 2.20E-11 |
| hsa_circ_0044224 | circRNA | 21.94 | 2.23E-10 |
| hsa_circ_0044825 | circRNA | 22.38 | 9.82E-11 |
| hsa_circ_0045134 | circRNA | 22.97 | 3.37E-11 |
| hsa_circ_0045219 | circRNA | -23.66 | 8.07E-12 |
| hsa_circ_0045416 | circRNA | 21.26 | 8.27E-10 |
| hsa_circ_0047131 | circRNA | 23.14 | 2.72E-11 |
| hsa_circ_0047133 | circRNA | 23.54 | 1.53E-11 |
| hsa_circ_0049285 | circRNA | 22.31 | 1.12E-10 |
| hsa_circ_0049288 | circRNA | 22.16 | 1.48E-10 |
| hsa_circ_0049941 | circRNA | 22.75 | 3.47E-11 |
| hsa_circ_0051030 | circRNA | 22.69 | 3.03E-11 |
| hsa_circ_0051031 | circRNA | 23.20 | 1.95E-11 |
| hsa_circ_0051039 | circRNA | 23.10 | 2.14E-11 |
| hsa_circ_0051040 | circRNA | 23.26 | 1.90E-11 |
| hsa_circ_0051333 | circRNA | 21.19 | 9.50E-10 |
| hsa_circ_0051395 | circRNA | 22.63 | 5.68E-11 |
| hsa_circ_0051396 | circRNA | 23.28 | 2.20E-11 |
| hsa_circ_0052544 | circRNA | 22.62 | 6.06E-11 |
| hsa_circ_0052545 | circRNA | 21.24 | 8.16E-10 |
| hsa_circ_0053905 | circRNA | 21.16 | 9.94E-10 |
| hsa_circ_0053906 | circRNA | 21.19 | 9.50E-10 |
| hsa_circ_0054171 | circRNA | -25.32 | 7.26E-13 |
| hsa_circ_0054172 | circRNA | -25.28 | 7.26E-13 |
| hsa_circ_0055149 | circRNA | -22.39 | 5.32E-11 |
| hsa_circ_0055363 | circRNA | 21.12 | 1.08E-09 |
| hsa_circ_0055591 | circRNA | 24.43 | 4.48E-12 |
| hsa_circ_0056007 | circRNA | 23.37 | 1.95E-11 |
| hsa_circ_0056575 | circRNA | -22.82 | 2.70E-11 |
| hsa_circ_0057795 | circRNA | 21.17 | 9.80E-10 |
| hsa_circ_0059045 | circRNA | 21.43 | 6.22E-10 |
| hsa_circ_0059049 | circRNA | 24.71 | 2.94E-12 |
| hsa_circ_0059051 | circRNA | 25.59 | 7.66E-13 |
| hsa_circ_0059056 | circRNA | 24.78 | 2.56E-12 |
| hsa_circ_0059057 | circRNA | 24.58 | 3.60E-12 |
| hsa_circ_0059576 | circRNA | 22.54 | 6.95E-11 |
| hsa_circ_0059577 | circRNA | 21.95 | 2.25E-10 |
| hsa_circ_0060534 | circRNA | 21.52 | 5.43E-10 |
| hsa_circ_0061677 | circRNA | 22.02 | 1.94E-10 |
| hsa_circ_0061678 | circRNA | 21.96 | 2.20E-10 |
| hsa_circ_0061704 | circRNA | -23.68 | 8.07E-12 |
| hsa_circ_0063153 | circRNA | 21.01 | 1.35E-09 |
| hsa_circ_0063227 | circRNA | 21.16 | 1.00E-09 |
| hsa_circ_0064022 | circRNA | 23.07 | 3.02E-11 |
| hsa_circ_0066259 | circRNA | 21.56 | 4.93E-10 |
| hsa_circ_0066770 | circRNA | 21.52 | 5.22E-10 |
| hsa_circ_0067471 | circRNA | 23.38 | 1.95E-11 |
| hsa_circ_0067504 | circRNA | 23.46 | 1.73E-11 |
| hsa_circ_0068100 | circRNA | 21.39 | 6.20E-10 |
| hsa_circ_0068642 | circRNA | 21.03 | 1.30E-09 |
| hsa_circ_0068759 | circRNA | 21.00 | 1.31E-09 |
| hsa_circ_0068760 | circRNA | 21.93 | 2.35E-10 |
| hsa_circ_0069819 | circRNA | 22.18 | 1.43E-10 |
| hsa_circ_0070660 | circRNA | 21.79 | 2.92E-10 |
| hsa_circ_0071176 | circRNA | 21.85 | 2.81E-10 |
| hsa_circ_0072308 | circRNA | 22.02 | 1.94E-10 |
| hsa_circ_0072932 | circRNA | 24.36 | 5.14E-12 |
| hsa_circ_0072939 | circRNA | 23.21 | 2.48E-11 |
| hsa_circ_0072940 | circRNA | 24.66 | 3.20E-12 |
| hsa_circ_0072942 | circRNA | 24.66 | 3.20E-12 |
| hsa_circ_0074831 | circRNA | -22.43 | 4.98E-11 |
| hsa_circ_0074855 | circRNA | 21.23 | 8.82E-10 |
| hsa_circ_0075026 | circRNA | 21.57 | 4.87E-10 |
| hsa_circ_0075607 | circRNA | 22.14 | 1.48E-10 |
| hsa_circ_0075608 | circRNA | 21.43 | 6.22E-10 |
| hsa_circ_0076168 | circRNA | 21.62 | 4.36E-10 |
| hsa_circ_0076175 | circRNA | 22.84 | 4.17E-11 |
| hsa_circ_0076377 | circRNA | -22.52 | 4.24E-11 |
| hsa_circ_0077705 | circRNA | -22.40 | 5.18E-11 |
| hsa_circ_0078017 | circRNA | -23.47 | 1.00E-11 |
| hsa_circ_0078205 | circRNA | 22.05 | 1.84E-10 |
| hsa_circ_0078363 | circRNA | 21.00 | 1.38E-09 |
| hsa_circ_0078364 | circRNA | 22.27 | 1.20E-10 |
| hsa_circ_0078373 | circRNA | 21.51 | 5.48E-10 |
| hsa_circ_0078911 | circRNA | -22.79 | 2.76E-11 |
| hsa_circ_0079287 | circRNA | 21.30 | 7.83E-10 |
| hsa_circ_0081560 | circRNA | 21.16 | 9.94E-10 |
| hsa_circ_0082958 | circRNA | 22.21 | 1.35E-10 |
| hsa_circ_0083054 | circRNA | 23.70 | 1.24E-11 |
| hsa_circ_0083455 | circRNA | 22.96 | 3.47E-11 |
| hsa_circ_0084115 | circRNA | 21.47 | 5.88E-10 |
| hsa_circ_0084546 | circRNA | 23.00 | 3.28E-11 |
| hsa_circ_0084581 | circRNA | 21.34 | 7.37E-10 |
| hsa_circ_0084678 | circRNA | 21.19 | 9.50E-10 |
| hsa_circ_0085280 | circRNA | 22.47 | 8.01E-11 |
| hsa_circ_0085326 | circRNA | 24.09 | 7.77E-12 |
| hsa_circ_0085596 | circRNA | 22.94 | 2.67E-11 |
| hsa_circ_0086186 | circRNA | 21.56 | 4.97E-10 |
| hsa_circ_0086462 | circRNA | 22.70 | 3.38E-11 |
| hsa_circ_0086736 | circRNA | 23.85 | 5.84E-13 |
| hsa_circ_0087127 | circRNA | 21.44 | 6.16E-10 |
| hsa_circ_0087212 | circRNA | 21.71 | 3.74E-10 |
| hsa_circ_0087619 | circRNA | 23.26 | 2.26E-11 |
| hsa_circ_0087621 | circRNA | 23.05 | 3.08E-11 |
| hsa_circ_0087624 | circRNA | 21.23 | 8.27E-10 |
| hsa_circ_0088025 | circRNA | 22.37 | 9.97E-11 |
| hsa_circ_0091577 | circRNA | -22.86 | 2.50E-11 |
| hsa_circ_0092513 | circRNA | 21.49 | 5.47E-10 |
| hsa_circ_0092849 | circRNA | 21.50 | 5.59E-10 |
| hsa_circ_0093336 | circRNA | 21.72 | 3.67E-10 |
| hsa_circ_0093523 | circRNA | -22.40 | 5.17E-11 |
| hsa_circ_0093590 | circRNA | -22.89 | 2.39E-11 |
| hsa_circ_0093788 | circRNA | 20.75 | 2.06E-09 |
| hsa_circ_0094015 | circRNA | 21.84 | 2.82E-10 |
| hsa_circ_0094019 | circRNA | 20.92 | 1.51E-09 |
| hsa_circ_0095221 | circRNA | 21.94 | 2.30E-10 |
| hsa_circ_0096371 | circRNA | 24.06 | 8.07E-12 |
| hsa_circ_0097161 | circRNA | 20.72 | 2.35E-09 |
| hsa_circ_0097344 | circRNA | 21.30 | 7.44E-10 |
| hsa_circ_0098611 | circRNA | 23.04 | 3.09E-11 |
| hsa_circ_0098863 | circRNA | -23.50 | 9.77E-12 |
| hsa_circ_0099781 | circRNA | -23.13 | 1.69E-11 |
| hsa_circ_0100476 | circRNA | 21.84 | 2.84E-10 |
| hsa_circ_0101695 | circRNA | 21.50 | 5.53E-10 |
| hsa_circ_0101735 | circRNA | 21.71 | 1.84E-10 |
| hsa_circ_0102561 | circRNA | -23.01 | 2.00E-11 |
| hsa_circ_0102601 | circRNA | 21.45 | 6.12E-10 |
| hsa_circ_0102633 | circRNA | 24.09 | 7.77E-12 |
| hsa_circ_0102741 | circRNA | 22.60 | 6.17E-11 |
| hsa_circ_0102925 | circRNA | -22.59 | 3.72E-11 |
| hsa_circ_0103341 | circRNA | 21.47 | 5.87E-10 |
| hsa_circ_0104801 | circRNA | 21.82 | 2.89E-10 |
| hsa_circ_0104802 | circRNA | 21.75 | 3.25E-10 |
| hsa_circ_0104803 | circRNA | 22.24 | 1.27E-10 |
| hsa_circ_0106283 | circRNA | 21.73 | 3.59E-10 |
| hsa_circ_0106598 | circRNA | 22.94 | 3.47E-11 |
| hsa_circ_0106752 | circRNA | 22.27 | 1.19E-10 |
| hsa_circ_0106816 | circRNA | 23.10 | 2.90E-11 |
| hsa_circ_0106862 | circRNA | 21.57 | 4.90E-10 |
| hsa_circ_0106969 | circRNA | 21.73 | 3.59E-10 |
| hsa_circ_0106972 | circRNA | 22.76 | 4.69E-11 |
| hsa_circ_0107080 | circRNA | 21.07 | 1.16E-09 |
| hsa_circ_0107154 | circRNA | -22.82 | 2.68E-11 |
| hsa_circ_0107244 | circRNA | 21.05 | 1.25E-09 |
| hsa_circ_0107245 | circRNA | 22.31 | 1.12E-10 |
| hsa_circ_0107246 | circRNA | 20.75 | 2.06E-09 |
| hsa_circ_0107276 | circRNA | 22.88 | 3.95E-11 |
| hsa_circ_0107481 | circRNA | 21.23 | 8.27E-10 |
| hsa_circ_0107484 | circRNA | 21.67 | 4.00E-10 |
| hsa_circ_0107533 | circRNA | 24.30 | 5.68E-12 |
| hsa_circ_0107534 | circRNA | 24.95 | 2.15E-12 |
| hsa_circ_0107717 | circRNA | 21.34 | 7.37E-10 |
| hsa_circ_0107998 | circRNA | -24.44 | 2.34E-12 |
| hsa_circ_0108056 | circRNA | 23.53 | 1.55E-11 |
| hsa_circ_0108060 | circRNA | 21.22 | 9.00E-10 |
| hsa_circ_0108063 | circRNA | 22.50 | 7.28E-11 |
| hsa_circ_0108161 | circRNA | 23.16 | 2.65E-11 |
| hsa_circ_0108730 | circRNA | 20.75 | 2.16E-09 |
| hsa_circ_0108731 | circRNA | 20.13 | 7.74E-09 |
| hsa_circ_0109818 | circRNA | 21.35 | 7.30E-10 |
| hsa_circ_0109923 | circRNA | -23.22 | 1.42E-11 |
| hsa_circ_0110198 | circRNA | -23.46 | 1.01E-11 |
| hsa_circ_0110892 | circRNA | 21.27 | 8.27E-10 |
| hsa_circ_0111109 | circRNA | 22.77 | 4.65E-11 |
| hsa_circ_0111361 | circRNA | -25.02 | 9.32E-13 |
| hsa_circ_0111495 | circRNA | 21.14 | 1.04E-09 |
| hsa_circ_0111496 | circRNA | 21.27 | 8.24E-10 |
| hsa_circ_0112150 | circRNA | 21.26 | 7.76E-10 |
| hsa_circ_0112954 | circRNA | -22.47 | 4.59E-11 |
| hsa_circ_0112968 | circRNA | 20.33 | 5.07E-09 |
| hsa_circ_0112969 | circRNA | 22.17 | 1.46E-10 |
| hsa_circ_0112971 | circRNA | 21.95 | 2.24E-10 |
| hsa_circ_0112999 | circRNA | 21.27 | 8.19E-10 |
| hsa_circ_0113903 | circRNA | 21.51 | 5.47E-10 |
| hsa_circ_0113950 | circRNA | 22.56 | 6.79E-11 |
| hsa_circ_0114351 | circRNA | 23.03 | 3.11E-11 |
| hsa_circ_0114376 | circRNA | -22.40 | 5.17E-11 |
| hsa_circ_0114830 | circRNA | 22.71 | 5.17E-11 |
| hsa_circ_0115430 | circRNA | 21.17 | 9.80E-10 |
| hsa_circ_0115813 | circRNA | 21.08 | 1.15E-09 |
| hsa_circ_0116309 | circRNA | -22.67 | 3.33E-11 |
| hsa_circ_0116310 | circRNA | -22.95 | 2.20E-11 |
| hsa_circ_0116314 | circRNA | -22.86 | 2.50E-11 |
| hsa_circ_0116392 | circRNA | 23.66 | 1.32E-11 |
| hsa_circ_0116525 | circRNA | 22.51 | 7.39E-11 |
| hsa_circ_0117007 | circRNA | 23.31 | 2.14E-11 |
| hsa_circ_0117307 | circRNA | 21.34 | 7.37E-10 |
| hsa_circ_0117395 | circRNA | 23.38 | 1.95E-11 |
| hsa_circ_0119852 | circRNA | -23.03 | 1.95E-11 |
| hsa_circ_0119932 | circRNA | 22.59 | 6.02E-11 |
| hsa_circ_0121488 | circRNA | 21.97 | 2.16E-10 |
| hsa_circ_0121790 | circRNA | -23.48 | 1.00E-11 |
| hsa_circ_0121791 | circRNA | -23.03 | 1.95E-11 |
| hsa_circ_0121792 | circRNA | -23.15 | 1.60E-11 |
| hsa_circ_0122056 | circRNA | 24.03 | 8.24E-12 |
| hsa_circ_0122081 | circRNA | 21.27 | 8.19E-10 |
| hsa_circ_0122092 | circRNA | 21.55 | 4.93E-10 |
| hsa_circ_0122146 | circRNA | -23.46 | 1.01E-11 |
| hsa_circ_0122150 | circRNA | -23.58 | 8.66E-12 |
| hsa_circ_0122581 | circRNA | 21.44 | 6.17E-10 |
| hsa_circ_0123284 | circRNA | 21.53 | 5.31E-10 |
| hsa_circ_0123500 | circRNA | 21.79 | 2.93E-10 |
| hsa_circ_0123777 | circRNA | -22.66 | 3.33E-11 |
| hsa_circ_0123889 | circRNA | 20.73 | 2.16E-09 |
| hsa_circ_0123890 | circRNA | 21.98 | 2.15E-10 |
| hsa_circ_0123911 | circRNA | 22.06 | 1.81E-10 |
| hsa_circ_0123992 | circRNA | 21.51 | 5.47E-10 |
| hsa_circ_0124193 | circRNA | -23.12 | 1.70E-11 |
| hsa_circ_0125195 | circRNA | 21.01 | 1.34E-09 |
| hsa_circ_0126251 | circRNA | 22.60 | 6.17E-11 |
| hsa_circ_0126490 | circRNA | 21.27 | 7.96E-10 |
| hsa_circ_0128319 | circRNA | -22.61 | 3.58E-11 |
| hsa_circ_0128435 | circRNA | 24.16 | 7.14E-12 |
| hsa_circ_0128436 | circRNA | 23.79 | 1.06E-11 |
| hsa_circ_0128596 | circRNA | 22.85 | 4.12E-11 |
| hsa_circ_0129047 | circRNA | 20.80 | 1.84E-09 |
| hsa_circ_0129303 | circRNA | 23.35 | 2.02E-11 |
| hsa_circ_0129307 | circRNA | 22.98 | 3.35E-11 |
| hsa_circ_0129322 | circRNA | 21.87 | 2.69E-10 |
| hsa_circ_0129323 | circRNA | 21.28 | 7.85E-10 |
| hsa_circ_0129342 | circRNA | -22.80 | 2.75E-11 |
| hsa_circ_0129343 | circRNA | -22.89 | 2.41E-11 |
| hsa_circ_0130059 | circRNA | 22.05 | 1.84E-10 |
| hsa_circ_0130288 | circRNA | 21.69 | 3.85E-10 |
| hsa_circ_0131124 | circRNA | 22.07 | 1.81E-10 |
| hsa_circ_0131402 | circRNA | 21.43 | 5.87E-10 |
| hsa_circ_0131403 | circRNA | 22.69 | 5.27E-11 |
| hsa_circ_0132148 | circRNA | 21.19 | 9.50E-10 |
| hsa_circ_0132355 | circRNA | 22.09 | 1.73E-10 |
| hsa_circ_0135742 | circRNA | 23.81 | 1.02E-11 |
| hsa_circ_0135744 | circRNA | 21.34 | 7.37E-10 |
| hsa_circ_0136911 | circRNA | 22.42 | 4.59E-11 |
| hsa_circ_0137830 | circRNA | 22.42 | 9.06E-11 |
| hsa_circ_0137832 | circRNA | 23.67 | 1.32E-11 |
| hsa_circ_0138195 | circRNA | 21.08 | 1.16E-09 |
| hsa_circ_0138380 | circRNA | 22.39 | 4.77E-11 |
| hsa_circ_0138632 | circRNA | 21.42 | 6.33E-10 |
| hsa_circ_0139129 | circRNA | 22.36 | 1.01E-10 |
| hsa_circ_0139131 | circRNA | 22.72 | 5.12E-11 |
| hsa_circ_0139687 | circRNA | 22.93 | 3.54E-11 |
