# Supplementary material for: Combining single-cell RNA sequencing of peripheral blood mononuclear cells and exosomal transcriptome to reveal the cellular and genetic profiles in COPD
Source: Respir Res. 2022 Sep 20;23:260. doi: 10.1186/s12931-022-02182-8 (PMC9490964; doi:10.1186/s12931-022-02182-8)
Supplement: Supplementary file 4 — Additional file 4: Table S5. Pairs of mRNA-miRNA predicted by TargetScan. Table S6. Pairs of lncRNA-miRNA predicted by miRcode. Table S7. Pairs of circRNA-miRNA predicted by starBase. [file 12931_2022_2182_MOESM4_ESM.docx]

Table S5. Pairs of mRNA-miRNA predicted by TargetScan.

| mRNA | miRNA |
| --- | --- |
| ZNF200 | hsa-let-7a-5p |
| CCR7 | hsa-let-7a-5p |
| ZNF200 | hsa-let-7b-5p |
| CCR7 | hsa-let-7b-5p |
| ZNF200 | hsa-let-7c-5p |
| CCR7 | hsa-let-7c-5p |
| ZNF200 | hsa-let-7d-5p |
| CCR7 | hsa-let-7d-5p |
| ZNF200 | hsa-let-7e-5p |
| CCR7 | hsa-let-7e-5p |
| ZNF200 | hsa-let-7f-5p |
| CCR7 | hsa-let-7f-5p |
| ZNF200 | hsa-let-7g-5p |
| CCR7 | hsa-let-7g-5p |
| ZNF200 | hsa-let-7i-5p |
| CCR7 | hsa-let-7i-5p |
| MRGBP | hsa-miR-101-3p |
| HEG1 | hsa-miR-101-3p |
| NPAS2 | hsa-miR-103a-3p |
| GNS | hsa-miR-106a-5p |
| NPAS2 | hsa-miR-106a-5p |
| HEG1 | hsa-miR-106a-5p |
| CXCL8 | hsa-miR-106a-5p |
| GNS | hsa-miR-106b-5p |
| NPAS2 | hsa-miR-106b-5p |
| HEG1 | hsa-miR-106b-5p |
| CXCL8 | hsa-miR-106b-5p |
| NPAS2 | hsa-miR-107 |
| CD151 | hsa-miR-124-3p |
| AREL1 | hsa-miR-125a-5p |
| AREL1 | hsa-miR-125b-5p |
| ITGA5 | hsa-miR-128-3p |
| ITGA5 | hsa-miR-1297 |
| CARMIL1 | hsa-miR-1297 |
| KIAA1217 | hsa-miR-130a-3p |
| CD69 | hsa-miR-130a-3p |
| RBBP8 | hsa-miR-130a-3p |
| SLC24A3 | hsa-miR-130a-3p |
| KIAA1217 | hsa-miR-130b-3p |
| CD69 | hsa-miR-130b-3p |
| RBBP8 | hsa-miR-130b-3p |
| SLC24A3 | hsa-miR-130b-3p |
| AP1AR | hsa-miR-133a-3p |
| AP1AR | hsa-miR-133b |
| CD69 | hsa-miR-137 |
| SLC24A3 | hsa-miR-137 |
| MRGBP | hsa-miR-137 |
| HEG1 | hsa-miR-139-5p |
| HEG1 | hsa-miR-140-5p |
| OSTM1 | hsa-miR-140-5p |
| FBXO3 | hsa-miR-142-3p |
| MBD6 | hsa-miR-142-3p |
| GNS | hsa-miR-143-3p |
| MRGBP | hsa-miR-144-3p |
| AREL1 | hsa-miR-145-5p |
| MRGBP | hsa-miR-145-5p |
| CARMIL1 | hsa-miR-145-5p |
| KIAA1217 | hsa-miR-148a-3p |
| ITGA5 | hsa-miR-148a-3p |
| ZNF274 | hsa-miR-148a-3p |
| KIAA1217 | hsa-miR-148b-3p |
| ITGA5 | hsa-miR-148b-3p |
| ZNF274 | hsa-miR-148b-3p |
| SGTA | hsa-miR-149-5p |
| MBD6 | hsa-miR-150-5p |
| KIAA1217 | hsa-miR-152-3p |
| ITGA5 | hsa-miR-152-3p |
| ZNF274 | hsa-miR-152-3p |
| SRPK1 | hsa-miR-153-3p |
| SRPK1 | hsa-miR-15a-5p |
| SRPK1 | hsa-miR-15b-5p |
| SRPK1 | hsa-miR-16-5p |
| GNS | hsa-miR-17-5p |
| NPAS2 | hsa-miR-17-5p |
| HEG1 | hsa-miR-17-5p |
| CXCL8 | hsa-miR-17-5p |
| ZNF200 | hsa-miR-181a-5p |
| ZNF514 | hsa-miR-181a-5p |
| ABI3BP | hsa-miR-181a-5p |
| ZNF200 | hsa-miR-181b-5p |
| ZNF514 | hsa-miR-181b-5p |
| ABI3BP | hsa-miR-181b-5p |
| ZNF514 | hsa-miR-181c-5p |
| ABI3BP | hsa-miR-181c-5p |
| ZNF200 | hsa-miR-181d-5p |
| ZNF514 | hsa-miR-181d-5p |
| ABI3BP | hsa-miR-181d-5p |
| AREL1 | hsa-miR-182-5p |
| SRPK1 | hsa-miR-186-5p |
| RBBP8 | hsa-miR-18a-5p |
| RBBP8 | hsa-miR-18b-5p |
| SRPK1 | hsa-miR-195-5p |
| SLC24A3 | hsa-miR-199a-5p |
| CDCA7L | hsa-miR-199a-5p |
| SLC24A3 | hsa-miR-199b-5p |
| CDCA7L | hsa-miR-199b-5p |
| B3GALNT2 | hsa-miR-19a-3p |
| KIAA1217 | hsa-miR-19a-3p |
| MBD6 | hsa-miR-19a-3p |
| RBBP8 | hsa-miR-19a-3p |
| NPAS2 | hsa-miR-19a-3p |
| SLC24A3 | hsa-miR-19a-3p |
| CARMIL1 | hsa-miR-19a-3p |
| B3GALNT2 | hsa-miR-19b-3p |
| KIAA1217 | hsa-miR-19b-3p |
| MBD6 | hsa-miR-19b-3p |
| RBBP8 | hsa-miR-19b-3p |
| NPAS2 | hsa-miR-19b-3p |
| SLC24A3 | hsa-miR-19b-3p |
| CARMIL1 | hsa-miR-19b-3p |
| OSTM1 | hsa-miR-200b-3p |
| OSTM1 | hsa-miR-200c-3p |
| AP1AR | hsa-miR-205-5p |
| GNS | hsa-miR-20a-5p |
| NPAS2 | hsa-miR-20a-5p |
| HEG1 | hsa-miR-20a-5p |
| CXCL8 | hsa-miR-20a-5p |
| GNS | hsa-miR-20b-5p |
| NPAS2 | hsa-miR-20b-5p |
| HEG1 | hsa-miR-20b-5p |
| CXCL8 | hsa-miR-20b-5p |
| CCR7 | hsa-miR-21-5p |
| AP1AR | hsa-miR-21-5p |
| CNOT8 | hsa-miR-21-5p |
| MBD6 | hsa-miR-218-5p |
| CARMIL1 | hsa-miR-22-3p |
| OSTM1 | hsa-miR-221-3p |
| OSTM1 | hsa-miR-222-3p |
| F2RL1 | hsa-miR-224-5p |
| SRPK1 | hsa-miR-23a-3p |
| SRPK1 | hsa-miR-23b-3p |
| MBD6 | hsa-miR-24-3p |
| AREL1 | hsa-miR-24-3p |
| CD69 | hsa-miR-25-3p |
| ITGA5 | hsa-miR-25-3p |
| GNS | hsa-miR-25-3p |
| SLC24A3 | hsa-miR-25-3p |
| AP1AR | hsa-miR-25-3p |
| CDCA7L | hsa-miR-25-3p |
| ITGA5 | hsa-miR-26a-5p |
| CARMIL1 | hsa-miR-26a-5p |
| ITGA5 | hsa-miR-26b-5p |
| CARMIL1 | hsa-miR-26b-5p |
| ITGA5 | hsa-miR-27a-3p |
| GNS | hsa-miR-27a-3p |
| HEG1 | hsa-miR-27a-3p |
| ITGA5 | hsa-miR-27b-3p |
| GNS | hsa-miR-27b-3p |
| HEG1 | hsa-miR-27b-3p |
| CDCA7L | hsa-miR-28-5p |
| GNS | hsa-miR-29a-3p |
| CNOT8 | hsa-miR-29a-3p |
| GNS | hsa-miR-29b-3p |
| CNOT8 | hsa-miR-29b-3p |
| GNS | hsa-miR-29c-3p |
| CNOT8 | hsa-miR-29c-3p |
| ABI3BP | hsa-miR-300 |
| AP1AR | hsa-miR-300 |
| KIAA1217 | hsa-miR-301a-3p |
| CD69 | hsa-miR-301a-3p |
| RBBP8 | hsa-miR-301a-3p |
| SLC24A3 | hsa-miR-301a-3p |
| KIAA1217 | hsa-miR-301b-3p |
| CD69 | hsa-miR-301b-3p |
| RBBP8 | hsa-miR-301b-3p |
| SLC24A3 | hsa-miR-301b-3p |
| GNS | hsa-miR-302a-3p |
| CXCL8 | hsa-miR-302a-3p |
| GNS | hsa-miR-302b-3p |
| CXCL8 | hsa-miR-302b-3p |
| GNS | hsa-miR-302c-3p |
| CXCL8 | hsa-miR-302c-3p |
| GNS | hsa-miR-302d-3p |
| CXCL8 | hsa-miR-302d-3p |
| GNS | hsa-miR-302e |
| CXCL8 | hsa-miR-302e |
| ZNF200 | hsa-miR-30a-5p |
| OSTM1 | hsa-miR-30a-5p |
| ZNF200 | hsa-miR-30b-5p |
| OSTM1 | hsa-miR-30b-5p |
| ZNF200 | hsa-miR-30c-5p |
| OSTM1 | hsa-miR-30c-5p |
| ZNF200 | hsa-miR-30d-5p |
| OSTM1 | hsa-miR-30d-5p |
| ZNF200 | hsa-miR-30e-5p |
| OSTM1 | hsa-miR-30e-5p |
| CD69 | hsa-miR-32-5p |
| ITGA5 | hsa-miR-32-5p |
| GNS | hsa-miR-32-5p |
| SLC24A3 | hsa-miR-32-5p |
| AP1AR | hsa-miR-32-5p |
| CDCA7L | hsa-miR-32-5p |
| ITGA5 | hsa-miR-326 |
| ITGA5 | hsa-miR-328-3p |
| ITGA5 | hsa-miR-330-5p |
| FBXO3 | hsa-miR-340-5p |
| F2RL1 | hsa-miR-340-5p |
| KIAA1217 | hsa-miR-34a-5p |
| MBD6 | hsa-miR-34a-5p |
| KIAA1217 | hsa-miR-34c-5p |
| MBD6 | hsa-miR-34c-5p |
| CD69 | hsa-miR-363-3p |
| ITGA5 | hsa-miR-363-3p |
| GNS | hsa-miR-363-3p |
| SLC24A3 | hsa-miR-363-3p |
| AP1AR | hsa-miR-363-3p |
| CDCA7L | hsa-miR-363-3p |
| CD69 | hsa-miR-367-3p |
| ITGA5 | hsa-miR-367-3p |
| GNS | hsa-miR-367-3p |
| SLC24A3 | hsa-miR-367-3p |
| AP1AR | hsa-miR-367-3p |
| CDCA7L | hsa-miR-367-3p |
| IL18BP | hsa-miR-370-3p |
| GNS | hsa-miR-372-3p |
| CXCL8 | hsa-miR-372-3p |
| GNS | hsa-miR-373-3p |
| CXCL8 | hsa-miR-373-3p |
| HEG1 | hsa-miR-374a-5p |
| CNOT8 | hsa-miR-374a-5p |
| HEG1 | hsa-miR-374b-5p |
| CNOT8 | hsa-miR-374b-5p |
| ABI3BP | hsa-miR-381-3p |
| AP1AR | hsa-miR-381-3p |
| SRPK1 | hsa-miR-424-5p |
| GNS | hsa-miR-425-5p |
| OSTM1 | hsa-miR-429 |
| KIAA1217 | hsa-miR-449a |
| MBD6 | hsa-miR-449a |
| KIAA1217 | hsa-miR-449b-5p |
| MBD6 | hsa-miR-449b-5p |
| KIAA1217 | hsa-miR-454-3p |
| CD69 | hsa-miR-454-3p |
| RBBP8 | hsa-miR-454-3p |
| SLC24A3 | hsa-miR-454-3p |
| THTPA | hsa-miR-485-5p |
| AP1AR | hsa-miR-488-3p |
| MBD6 | hsa-miR-490-3p |
| SART3 | hsa-miR-494-3p |
| NPAS2 | hsa-miR-494-3p |
| SRPK1 | hsa-miR-497-5p |
| GNS | hsa-miR-519d-3p |
| NPAS2 | hsa-miR-519d-3p |
| HEG1 | hsa-miR-519d-3p |
| CXCL8 | hsa-miR-519d-3p |
| GNS | hsa-miR-520a-3p |
| CXCL8 | hsa-miR-520a-3p |
| GNS | hsa-miR-520b |
| CXCL8 | hsa-miR-520b |
| GNS | hsa-miR-520c-3p |
| CXCL8 | hsa-miR-520c-3p |
| GNS | hsa-miR-520d-3p |
| CXCL8 | hsa-miR-520d-3p |
| GNS | hsa-miR-520e |
| CXCL8 | hsa-miR-520e |
| HEG1 | hsa-miR-543 |
| CCR7 | hsa-miR-590-5p |
| AP1AR | hsa-miR-590-5p |
| CNOT8 | hsa-miR-590-5p |
| CNOT8 | hsa-miR-7-5p |
| CDCA7L | hsa-miR-708-5p |
| GNS | hsa-miR-873-5p |
| CMTM7 | hsa-miR-873-5p |
| OSTM1 | hsa-miR-874-3p |
| CD69 | hsa-miR-92a-3p |
| ITGA5 | hsa-miR-92a-3p |
| GNS | hsa-miR-92a-3p |
| SLC24A3 | hsa-miR-92a-3p |
| AP1AR | hsa-miR-92a-3p |
| CDCA7L | hsa-miR-92a-3p |
| CD69 | hsa-miR-92b-3p |
| ITGA5 | hsa-miR-92b-3p |
| GNS | hsa-miR-92b-3p |
| SLC24A3 | hsa-miR-92b-3p |
| AP1AR | hsa-miR-92b-3p |
| CDCA7L | hsa-miR-92b-3p |
| GNS | hsa-miR-93-5p |
| NPAS2 | hsa-miR-93-5p |
| HEG1 | hsa-miR-93-5p |
| CXCL8 | hsa-miR-93-5p |
| ZNF200 | hsa-miR-98-5p |
| CCR7 | hsa-miR-98-5p |

Table S6. Pairs of lncRNA-miRNA predicted by miRcode.

| lncRNA | miRNA |
| --- | --- |
| LINC00324 | hsa-miR-9 |
| LINC00324 | hsa-miR-9ab |
| LINC00324 | hsa-miR-139-5p |
| LINC00324 | hsa-miR-141 |
| LINC00324 | hsa-miR-200a |
| LINC00324 | hsa-miR-142-3p |
| LINC00324 | hsa-miR-143 |
| LINC00324 | hsa-miR-1721 |
| LINC00324 | hsa-miR-4770 |
| LINC00324 | hsa-miR-148ab-3p |
| LINC00324 | hsa-miR-152 |
| LINC00324 | hsa-miR-15abc |
| LINC00324 | hsa-miR-16 |
| LINC00324 | hsa-miR-16abc |
| LINC00324 | hsa-miR-195 |
| LINC00324 | hsa-miR-322 |
| LINC00324 | hsa-miR-424 |
| LINC00324 | hsa-miR-497 |
| LINC00324 | hsa-miR-1907 |
| LINC00324 | hsa-miR-214 |
| LINC00324 | hsa-miR-761 |
| LINC00324 | hsa-miR-3619-5p |
| LINC00324 | hsa-miR-216b |
| LINC00324 | hsa-miR-216b-5p |
| LINC00324 | hsa-miR-22 |
| LINC00324 | hsa-miR-22-3p |
| LINC00324 | hsa-miR-122 |
| LINC00324 | hsa-miR-122a |
| LINC00324 | hsa-miR-1352 |
| LINC00324 | hsa-miR-23abc |
| LINC00324 | hsa-miR-23b-3p |
| LINC00324 | hsa-miR-33a-3p |
| LINC00324 | hsa-miR-365 |
| LINC00324 | hsa-miR-365-3p |
| LINC00324 | hsa-miR-375 |
| LINC00324 | hsa-miR-129-5p |
| LINC00324 | hsa-miR-129ab-5p |
| LINC00324 | hsa-miR-490-3p |
| COL6A4P2 | hsa-miR-130ac |
| COL6A4P2 | hsa-miR-301ab |
| COL6A4P2 | hsa-miR-301b |
| COL6A4P2 | hsa-miR-301b-3p |
| COL6A4P2 | hsa-miR-454 |
| COL6A4P2 | hsa-miR-721 |
| COL6A4P2 | hsa-miR-4295 |
| COL6A4P2 | hsa-miR-3666 |
| COL6A4P2 | hsa-miR-132 |
| COL6A4P2 | hsa-miR-212 |
| COL6A4P2 | hsa-miR-212-3p |
| COL6A4P2 | hsa-miR-7 |
| COL6A4P2 | hsa-miR-7ab |
| COL6A4P2 | hsa-miR-133abc |
| COL6A4P2 | hsa-miR-93 |
| COL6A4P2 | hsa-miR-93a |
| COL6A4P2 | hsa-miR-105 |
| COL6A4P2 | hsa-miR-106a |
| COL6A4P2 | hsa-miR-291a-3p |
| COL6A4P2 | hsa-miR-294 |
| COL6A4P2 | hsa-miR-295 |
| COL6A4P2 | hsa-miR-302abcde |
| COL6A4P2 | hsa-miR-372 |
| COL6A4P2 | hsa-miR-373 |
| COL6A4P2 | hsa-miR-428 |
| COL6A4P2 | hsa-miR-519a |
| COL6A4P2 | hsa-miR-520be |
| COL6A4P2 | hsa-miR-520acd-3p |
| COL6A4P2 | hsa-miR-1378 |
| COL6A4P2 | hsa-miR-1420ac |
| COL6A4P2 | hsa-miR-96 |
| COL6A4P2 | hsa-miR-507 |
| COL6A4P2 | hsa-miR-1271 |
| COL6A4P2 | hsa-miR-137 |
| COL6A4P2 | hsa-miR-137ab |
| COL6A4P2 | hsa-miR-138 |
| COL6A4P2 | hsa-miR-138ab |
| COL6A4P2 | hsa-miR-142-3p |
| COL6A4P2 | hsa-miR-143 |
| COL6A4P2 | hsa-miR-1721 |
| COL6A4P2 | hsa-miR-4770 |
| COL6A4P2 | hsa-miR-144 |
| COL6A4P2 | hsa-miR-145 |
| COL6A4P2 | hsa-miR-148ab-3p |
| COL6A4P2 | hsa-miR-152 |
| COL6A4P2 | hsa-miR-150 |
| COL6A4P2 | hsa-miR-5127 |
| COL6A4P2 | hsa-miR-15abc |
| COL6A4P2 | hsa-miR-16 |
| COL6A4P2 | hsa-miR-16abc |
| COL6A4P2 | hsa-miR-195 |
| COL6A4P2 | hsa-miR-322 |
| COL6A4P2 | hsa-miR-424 |
| COL6A4P2 | hsa-miR-497 |
| COL6A4P2 | hsa-miR-1907 |
| COL6A4P2 | hsa-miR-181abcd |
| COL6A4P2 | hsa-miR-4262 |
| COL6A4P2 | hsa-miR-182 |
| COL6A4P2 | hsa-miR-190 |
| COL6A4P2 | hsa-miR-190ab |
| COL6A4P2 | hsa-miR-194 |
| COL6A4P2 | hsa-miR-196abc |
| COL6A4P2 | hsa-miR-199ab-5p |
| COL6A4P2 | hsa-miR-1ab |
| COL6A4P2 | hsa-miR-206 |
| COL6A4P2 | hsa-miR-613 |
| COL6A4P2 | hsa-miR-204 |
| COL6A4P2 | hsa-miR-204b |
| COL6A4P2 | hsa-miR-211 |
| COL6A4P2 | hsa-miR-210 |
| COL6A4P2 | hsa-miR-214 |
| COL6A4P2 | hsa-miR-761 |
| COL6A4P2 | hsa-miR-3619-5p |
| COL6A4P2 | hsa-miR-216a |
| COL6A4P2 | hsa-miR-216b |
| COL6A4P2 | hsa-miR-216b-5p |
| COL6A4P2 | hsa-miR-22 |
| COL6A4P2 | hsa-miR-22-3p |
| COL6A4P2 | hsa-miR-221 |
| COL6A4P2 | hsa-miR-222 |
| COL6A4P2 | hsa-miR-222ab |
| COL6A4P2 | hsa-miR-1928 |
| COL6A4P2 | hsa-miR-23abc |
| COL6A4P2 | hsa-miR-23b-3p |
| COL6A4P2 | hsa-miR-26ab |
| COL6A4P2 | hsa-miR-1297 |
| COL6A4P2 | hsa-miR-4465 |
| COL6A4P2 | hsa-miR-29abcd |
| COL6A4P2 | hsa-miR-103a |
| COL6A4P2 | hsa-miR-107 |
| COL6A4P2 | hsa-miR-107ab |
| COL6A4P2 | hsa-miR-338 |
| COL6A4P2 | hsa-miR-338-3p |
| COL6A4P2 | hsa-miR-33a-3p |
| COL6A4P2 | hsa-miR-365 |
| COL6A4P2 | hsa-miR-365-3p |
| COL6A4P2 | hsa-miR-33ab |
| COL6A4P2 | hsa-miR-33-5p |
| COL6A4P2 | hsa-miR-34ac |
| COL6A4P2 | hsa-miR-34bc-5p |
| COL6A4P2 | hsa-miR-449abc |
| COL6A4P2 | hsa-miR-449c-5p |
| COL6A4P2 | hsa-miR-375 |
| COL6A4P2 | hsa-miR-425 |
| COL6A4P2 | hsa-miR-425-5p |
| COL6A4P2 | hsa-miR-489 |
| COL6A4P2 | hsa-miR-125a-5p |
| COL6A4P2 | hsa-miR-125b-5p |
| COL6A4P2 | hsa-miR-351 |
| COL6A4P2 | hsa-miR-670 |
| COL6A4P2 | hsa-miR-4319 |
| COL6A4P2 | hsa-miR-10abc |
| COL6A4P2 | hsa-miR-10a-5p |
| COL6A4P2 | hsa-miR-128 |
| COL6A4P2 | hsa-miR-128ab |

Table S7. Pairs of circRNA-miRNA predicted by starBase.

| circRNA | miRNA |
| --- | --- |
| hsa_circ_0000415 | hsa-miR-105-5p |
| hsa_circ_0000415 | hsa-miR-1185-5p |
| hsa_circ_0001769 | hsa-miR-125a-5p |
| hsa_circ_0001769 | hsa-miR-125b-5p |
| hsa_circ_0001333 | hsa-miR-1278 |
| hsa_circ_0000415 | hsa-miR-1278 |
| hsa_circ_0000466 | hsa-miR-1278 |
| hsa_circ_0001785 | hsa-miR-1306-5p |
| hsa_circ_0001365 | hsa-miR-130a-5p |
| hsa_circ_0001741 | hsa-miR-137 |
| hsa_circ_0001741 | hsa-miR-141-3p |
| hsa_circ_0001333 | hsa-miR-144-5p |
| hsa_circ_0001741 | hsa-miR-188-5p |
| hsa_circ_0001785 | hsa-miR-191-5p |
| hsa_circ_0001741 | hsa-miR-199a-5p |
| hsa_circ_0001741 | hsa-miR-199b-5p |
| hsa_circ_0001741 | hsa-miR-200a-3p |
| hsa_circ_0000415 | hsa-miR-2114-5p |
| hsa_circ_0001069 | hsa-miR-214-3p |
| hsa_circ_0000466 | hsa-miR-216a-5p |
| hsa_circ_0001741 | hsa-miR-224-5p |
| hsa_circ_0001789 | hsa-miR-2355-3p |
| hsa_circ_0001365 | hsa-miR-23a-3p |
| hsa_circ_0001365 | hsa-miR-23b-3p |
| hsa_circ_0001365 | hsa-miR-23c |
| hsa_circ_0001741 | hsa-miR-31-5p |
| hsa_circ_0001741 | hsa-miR-3150a-3p |
| hsa_circ_0001769 | hsa-miR-3150b-3p |
| hsa_circ_0001741 | hsa-miR-3163 |
| hsa_circ_0000415 | hsa-miR-330-3p |
| hsa_circ_0001069 | hsa-miR-345-3p |
| hsa_circ_0001069 | hsa-miR-3619-5p |
| hsa_circ_0000415 | hsa-miR-365a-3p |
| hsa_circ_0000415 | hsa-miR-365b-3p |
| hsa_circ_0000415 | hsa-miR-3679-5p |
| hsa_circ_0001789 | hsa-miR-376a-3p |
| hsa_circ_0001789 | hsa-miR-376b-3p |
| hsa_circ_0000740 | hsa-miR-382-3p |
| hsa_circ_0001769 | hsa-miR-4319 |
| hsa_circ_0000415 | hsa-miR-4712-5p |
| hsa_circ_0000415 | hsa-miR-4761-3p |
| hsa_circ_0001741 | hsa-miR-4766-3p |
| hsa_circ_0001769 | hsa-miR-4784 |
| hsa_circ_0001365 | hsa-miR-488-3p |
| hsa_circ_0001741 | hsa-miR-491-5p |
| hsa_circ_0000415 | hsa-miR-495-3p |
| hsa_circ_0001069 | hsa-miR-512-3p |
| hsa_circ_0001333 | hsa-miR-519a-3p |
| hsa_circ_0001333 | hsa-miR-519b-3p |
| hsa_circ_0001333 | hsa-miR-519c-3p |
| hsa_circ_0001741 | hsa-miR-545-3p |
| hsa_circ_0000415 | hsa-miR-545-5p |
| hsa_circ_0001741 | hsa-miR-552-3p |
| hsa_circ_0000415 | hsa-miR-5688 |
| hsa_circ_0000466 | hsa-miR-577 |
| hsa_circ_0001741 | hsa-miR-582-3p |
| hsa_circ_0000415 | hsa-miR-582-5p |
| hsa_circ_0001741 | hsa-miR-605-3p |
| hsa_circ_0001069 | hsa-miR-6509-3p |
| hsa_circ_0001069 | hsa-miR-670-5p |
| hsa_circ_0001769 | hsa-miR-670-5p |
| hsa_circ_0001741 | hsa-miR-6763-5p |
| hsa_circ_0001741 | hsa-miR-6866-3p |
| hsa_circ_0000740 | hsa-miR-7-5p |
| hsa_circ_0001069 | hsa-miR-761 |
| hsa_circ_0000415 | hsa-miR-770-5p |
| hsa_circ_0000415 | hsa-miR-7853-5p |
| hsa_circ_0001069 | hsa-miR-9-5p |
