# Supplementary material for: Combining single-cell RNA sequencing of peripheral blood mononuclear cells and exosomal transcriptome to reveal the cellular and genetic profiles in COPD
Source: Respir Res. 2022 Sep 20;23:260. doi: 10.1186/s12931-022-02182-8 (PMC9490964; doi:10.1186/s12931-022-02182-8)
Supplement: Supplementary file 6 — Additional file 6: Table S8. Single-cell differentially expressed mRNA in overall cells between COPD patient and healthy control. Table S9. Gene list for construction of protein–protein interaction network [file 12931_2022_2182_MOESM6_ESM.docx]

Table S8. Single-cell differentially expressed mRNA in overall cells between COPD patient and healthy control.

| Gene Symbol | Type | log2 (Disease / Normal) | padjust (Disease / Normal) |
| --- | --- | --- | --- |
| FGR | mRNA | 0.306864 | 1.65E-25 |
| CD79B | mRNA | -0.36132 | 6.12E-87 |
| PLAUR | mRNA | 0.685804 | 4.42E-97 |
| TYROBP | mRNA | 0.938741 | 3.32E-173 |
| SERPINB1 | mRNA | 0.276706 | 6.44E-133 |
| MAP2K3 | mRNA | 0.302356 | 1.59E-155 |
| VCAN | mRNA | 0.508858 | 1.54E-26 |
| TGFBR3 | mRNA | 0.259409 | 1.47E-25 |
| PTGS2 | mRNA | 1.051681 | 8.68E-10 |
| ACTB | mRNA | 0.349979 | 1.14E-146 |
| CST7 | mRNA | 0.580983 | 6.40E-194 |
| CXCL2 | mRNA | 0.43266 | 5.73E-111 |
| MEF2C | mRNA | -0.30298 | 2.26E-05 |
| GSTP1 | mRNA | 0.342764 | 5.48E-68 |
| APLP2 | mRNA | 0.342685 | 1.64E-91 |
| FCN1 | mRNA | 0.580658 | 5.29E-88 |
| LYZ | mRNA | 1.438485 | 2.05E-92 |
| LGALS1 | mRNA | 0.524072 | 5.04E-37 |
| IL2RB | mRNA | 0.295183 | 3.03E-22 |
| GZMH | mRNA | 0.44152 | 1.28E-14 |
| GZMB | mRNA | 0.650068 | 1.55E-43 |
| CST3 | mRNA | 0.637351 | 1.22E-133 |
| PYCARD | mRNA | 0.262246 | 4.04E-62 |
| CD37 | mRNA | -0.27293 | 4.71E-35 |
| FCER2 | mRNA | -0.27591 | 8.03E-197 |
| CD79A | mRNA | -0.34229 | 4.35E-105 |
| NKG7 | mRNA | 0.742153 | 9.57E-58 |
| NAMPT | mRNA | 0.378443 | 1.63E-178 |
| CPVL | mRNA | 0.257215 | 7.87E-21 |
| PTGDS | mRNA | 0.572473 | 0 |
| MAP3K8 | mRNA | 0.333075 | 8.10E-32 |
| PF4V1 | mRNA | 0.456031 | 0 |
| RPS13 | mRNA | -0.37754 | 9.56E-207 |
| IFNG | mRNA | 0.282942 | 1.21E-16 |
| GAPDH | mRNA | 0.254588 | 6.31E-65 |
| GNPTAB | mRNA | 0.267046 | 4.98E-19 |
| LDHB | mRNA | -0.25994 | 0.001085 |
| SOD2 | mRNA | 0.253931 | 2.35E-88 |
| RPS12 | mRNA | -0.35815 | 6.60E-290 |
| VEGFA | mRNA | 0.287932 | 3.45E-09 |
| GNLY | mRNA | 0.814098 | 1.22E-31 |
| ID2 | mRNA | 0.28843 | 4.90E-93 |
| RALGPS2 | mRNA | -0.42092 | 2.96E-129 |
| RNF19B | mRNA | 0.337272 | 1.35E-135 |
| CTSD | mRNA | 0.437611 | 2.73E-174 |
| CSF3R | mRNA | 0.56953 | 4.76E-106 |
| SRGN | mRNA | 0.550514 | 4.49E-159 |
| G0S2 | mRNA | 0.51836 | 1.00E-08 |
| EREG | mRNA | 0.507105 | 1.34E-40 |
| IL1B | mRNA | 0.64007 | 4.95E-42 |
| FGL2 | mRNA | 0.255594 | 0 |
| SAT1 | mRNA | 0.270306 | 3.03E-64 |
| LGALS3 | mRNA | 0.260984 | 1.27E-54 |
| PTPRE | mRNA | 0.433402 | 1.22E-184 |
| KLRD1 | mRNA | 0.568839 | 1.45E-13 |
| ANXA1 | mRNA | 0.266823 | 4.58E-19 |
| CD63 | mRNA | 0.321301 | 5.29E-75 |
| KLF4 | mRNA | 0.380522 | 1.99E-67 |
| IER3 | mRNA | 0.801202 | 6.48E-125 |
| FGFBP2 | mRNA | 0.521969 | 0.019996 |
| THBS1 | mRNA | 0.259493 | 1.59E-81 |
| RPLP1 | mRNA | -0.30439 | 1.53E-191 |
| LEF1 | mRNA | -0.25085 | 5.60E-271 |
| DUSP6 | mRNA | 0.279983 | 0 |
| BCL2A1 | mRNA | 0.396245 | 4.60E-40 |
| RPS8 | mRNA | -0.35766 | 0 |
| MCL1 | mRNA | 0.258926 | 3.57E-97 |
| S100A8 | mRNA | 1.06542 | 0 |
| AFF3 | mRNA | -0.31008 | 4.85E-233 |
| GZMA | mRNA | 0.550132 | 1.37E-39 |
| RPL10 | mRNA | -0.31807 | 2.18E-237 |
| KLRF1 | mRNA | 0.408072 | 2.88E-29 |
| BANK1 | mRNA | -0.3725 | 7.95E-79 |
| TTN | mRNA | -0.25727 | 0 |
| MS4A1 | mRNA | -0.53728 | 8.57E-120 |
| FCER1G | mRNA | 0.758727 | 6.56E-160 |
| SPON2 | mRNA | 0.509882 | 1.61E-04 |
| TNFRSF13C | mRNA | -0.27375 | 3.46E-124 |
| NLRP3 | mRNA | 0.422723 | 8.05E-27 |
| CTSS | mRNA | 0.645871 | 3.00E-80 |
| S100A11 | mRNA | 0.509979 | 5.62E-72 |
| S100A9 | mRNA | 1.357297 | 0 |
| S100A12 | mRNA | 0.413125 | 2.21E-67 |
| FCRL1 | mRNA | -0.26774 | 1.83E-47 |
| MNDA | mRNA | 0.506934 | 0.001032 |
| ANXA5 | mRNA | 0.27351 | 2.29E-80 |
| LAIR2 | mRNA | 0.300264 | 4.08E-174 |
| CX3CR1 | mRNA | 0.306184 | 0.004014 |
| CXCL8 | mRNA | 1.313399 | 2.17E-102 |
| CLIC3 | mRNA | 0.531251 | 7.74E-57 |
| ITGAM | mRNA | 0.286064 | 2.58E-43 |
| GABARAP | mRNA | 0.288696 | 2.06E-52 |
| JUNB | mRNA | -0.37946 | 1.93E-116 |
| HOPX | mRNA | 0.330148 | 2.81E-13 |
| MAL | mRNA | -0.28171 | 0 |
| CEBPB | mRNA | 0.28085 | 1.26E-151 |
| CLEC12A | mRNA | 0.25261 | 1.06E-191 |
| CTSW | mRNA | 0.453031 | 4.09E-59 |
| TRIB1 | mRNA | 0.285803 | 1.11E-222 |
| METRNL | mRNA | 0.30884 | 1.24E-05 |
| S1PR5 | mRNA | 0.284976 | 0.035701 |
| NPM1 | mRNA | -0.33795 | 2.22E-141 |
| ANXA2 | mRNA | 0.314825 | 4.74E-98 |
| MYBL1 | mRNA | 0.342298 | 1.14E-28 |
| RPS23 | mRNA | -0.39104 | 0 |
| CMC1 | mRNA | 0.378689 | 8.67E-31 |
| HBA2 | mRNA | -0.46206 | 0 |
| S100A4 | mRNA | 0.481798 | 4.41E-156 |
| DTHD1 | mRNA | 0.277254 | 7.38E-69 |
| SERPINA1 | mRNA | 0.342592 | 3.74E-21 |
| PSAP | mRNA | 0.485035 | 2.23E-136 |
| S100A10 | mRNA | 0.265331 | 1.19E-56 |
| CFD | mRNA | 0.275407 | 9.88E-189 |
| S100A6 | mRNA | 0.505006 | 6.98E-109 |
| CD247 | mRNA | 0.33766 | 8.43E-120 |
| RPL39 | mRNA | -0.35593 | 1.02E-232 |
| FCGR3A | mRNA | 0.539078 | 5.09E-07 |
| MAFB | mRNA | 0.289023 | 4.62E-218 |
| AIF1 | mRNA | 0.37226 | 3.94E-274 |
| ADGRG1 | mRNA | 0.332953 | 2.04E-19 |
| KLRC2 | mRNA | 0.329951 | 2.58E-189 |
| HBA1 | mRNA | -0.27054 | 9.85E-94 |
| IGKC | IG_C_gene | -0.84384 | 4.94E-77 |
| IGLC1 | IG_C_gene | -0.37283 | 6.94E-296 |
| IGLC2 | IG_C_gene | -0.64933 | 1.59E-244 |
| IGLC3 | IG_C_gene | -0.70749 | 1.13E-171 |
| TRDC | TR_C_gene | 0.454705 | 1.29E-175 |
| IGHD | IG_C_gene | -0.42205 | 2.54E-112 |
| IGHM | IG_C_gene | -0.60646 | 1.31E-294 |
| CEBPD | mRNA | 0.402089 | 5.05E-52 |
| LTB | mRNA | -0.59862 | 4.33E-175 |
| RPS18 | mRNA | -0.33264 | 0 |
| RPS28 | mRNA | -0.36462 | 7.70E-259 |
| C12orf75 | mRNA | 0.283344 | 6.54E-29 |
| CD302 | mRNA | 0.266214 | 3.64E-83 |
| HBB | mRNA | -1.13929 | 0 |
| CCL5 | mRNA | 0.530556 | 1.34E-131 |
| CCL4L2 | mRNA | 0.326267 | 8.40E-13 |
| CCL3L1 | mRNA | 0.373696 | 9.56E-42 |
| CCL3 | mRNA | 0.490041 | 5.03E-08 |

Table S9. Gene list for construction of PPI network

| Exosome Transcriptome | Target of lncRNA | Single-cell Transcriptome |
| --- | --- | --- |
| PIGZ | LOC107984832 | LYZ |
| NCBP2L | TMEM17 | S100A9 |
| THTPA | IFT52 | CXCL8 |
| LZTFL1 | GVQW3 | S100A8 |
| SMCP | ELOVL7 | PTGS2 |
| MARCHF11 | HLA-DQB1 | TYROBP |
| TAS2R8 | RNF135 | GNLY |
| PKP3 | AGAP9 | IER3 |
| OAS1 | SEZ6 | FCER1G |
| LOC728392 | SLC39A10 | NKG7 |
| TLDC2 | DENND1B | PLAUR |
| ZNF799 | ETNK1 | GZMB |
| CD151 | NCKAP1L | CTSS |
| ZNF274 | ZFP42 | IL1B |
| LPO | CTTN | CST3 |
| KBTBD3 | FAM83D | CST7 |
| GCOM1 | NOTCH2NLC | FCN1 |
| MRGBP | ZNF780A | PTGDS |
| C7orf31 | ARL17B | CSF3R |
| SPATA1 | SYT17 | KLRD1 |
| TRMO | BEST3 | SRGN |
| EPHB2 | FBXO17 | GZMA |
| SLPI | ZNF106 | FCGR3A |
| SNX20 | ZNF568 | CLIC3 |
| CMTM7 | ZBTB11 | CCL5 |
| ANO9 | PGPEP1 | LGALS1 |
| TNFSF8 | ANKK1 | FGFBP2 |
| ARID5A | IFIT5 | G0S2 |
| CLECL1 | HBS1L | S100A11 |
| DEFA4 | MAP3K2 | SPON2 |
| CD69 | PRKCQ | VCAN |
| SLC6A1 | ZNF571 | EREG |
| LSM5 | ZNF540 | MNDA |
| SEC11C | AGAP11 | S100A6 |
| CXCL11 | EGLN1 | CCL3 |
| SLC24A3 | SOCS4 | PSAP |
| LOC107986004 | ANKFN1 | S100A4 |
| DPEP2 | NBPF6 | PF4V1 |
| ZBED8 | BMS1 | TRDC |
| CLDN18 | ZAP70 | CTSW |
| RFPL4A | CCR4 | GZMH |
| CD79B | AKT3 | CTSD |
| SOX15 | LRRC53 | PTPRE |
| PPP1R35 | LRRC37A2 | CXCL2 |
| PM20D1 | WDR73 | NLRP3 |
| TNFRSF25 | CECR2 | S100A12 |
| CGAS | CYB5RL | KLRF1 |
| EFHC2 | SOCS7 | CEBPD |
| ROPN1 | MIB1 | BCL2A1 |
| TMEM155 | PPIF | KLF4 |
| ANKRD34C | LOC112267904 | CMC1 |
| PRSS36 | ITPRIPL2 | NAMPT |
| ZNF514 | PARP3 | CCL3L1 |
| CALML4 | MS4A10 | AIF1 |
| ZNF836 | FLRT2 | ACTB |
| SERPINE3 | JMY | GSTP1 |
| IFNG | SMC3 | APLP2 |
| KCNA7 | ANKRD9 | SERPINA1 |
| CD200R1 | LRRC37A3 | MYBL1 |
| IL18BP | YWHAQ | CD247 |
| CELF4 | HLA-DPA1 | RNF19B |
| ZNF200 | DNAH12 | MAP3K8 |
| CEACAM1 | NLRP11 | ADGRG1 |
| FBXO3 | RUNX1T1 | HOPX |
| ALOX5AP | PLPBP | KLRC2 |
| B3GALNT2 | ARL17A | CCL4L2 |
| PRR26 | RPS18 | CD63 |
| IMPG2 | LRRC37A | ANXA2 |
| YKT6 | NUS1 | METRNL |
| RBBP8 | PABPC1 | FGR |
| SGTA | FAM181B | CX3CR1 |
| PODN | RIPK3 | MAP2K3 |
| CCDC191 | HLA-DRA | LAIR2 |
| MRTFB | LOC107987125 | IL2RB |
| MGP | ZNF404 | MAFB |
| AMN1 | OR1J1 | GABARAP |
| HEG1 | LOC100996709 | ID2 |
| AP1AR | AGAP5 | VEGFA |
| CDCA7L | ZSWIM8 | ITGAM |
| CARMIL1 | ZFP64 | TRIB1 |
| SIPA1L3 | AGAP1 | S1PR5 |
| CKMT2 | GABRG2 | C12orf75 |
| MRPL39 | EGR3 | IFNG |
| DDRGK1 | FOXN4 | CEBPB |
| SRPK1 | SDCCAG8 | DUSP6 |
| MMP8 | EVA1C | DTHD1 |
| SART3 | KATNAL2 | SERPINB1 |
| AREL1 | SMO | CFD |
| CNOT8 | D2HGDH | ANXA5 |
| CXCL8 | NAALADL2 | SAT1 |
| HCAR3 | TBC1D20 | GNPTAB |
| OSTM1 | UVSSA | ANXA1 |
| KIAA1217 | LOC107984156 | CD302 |
| CTNNA2 | COL19A1 | S100A10 |
| FOXB1 | FKTN | PYCARD |
| GNS | ZMIZ1 | LGALS3 |
| ELAPOR1 | ZNF780B | THBS1 |
| ZNF768 | EXPH5 | TGFBR3 |
| ITGA5 | GCSAM | MCL1 |
| ABI3BP | ADCY4 | CPVL |
| FSIP2 | PROCA1 | FGL2 |
| SCGB1C2 | STON2 | GAPDH |
| CHRNB2 | ADAMTS7 | SOD2 |
| CHRNA10 | SLC35C1 | CLEC12A |
| MED20 | E2F3 | LEF1 |
| C12orf40 | POGZ | TTN |
| CTSG | TRIM2 | LDHB |
| DPH3P1 | ABCA8 | FCRL1 |
| PRF1 | HDGFL3 | HBA1 |
| NPAS2 | RNF130 | CD37 |
| F2RL1 | ACP7 | TNFRSF13C |
| PDE6G |  | FCER2 |
| C11orf21 |  | MAL |
| CCR7 |  | MEF2C |
| MBD6 |  | RPLP1 |
| SPTB |  | AFF3 |
| ITGB1BP2 |  | RPL10 |
| ADGRB2 |  | RPS18 |
| HP |  | NPM1 |
| FAM81B |  | CD79A |
| JAM2 |  | RPL39 |
| EPB42 |  | RPS8 |
| TCHHL1 |  | RPS12 |
| GFAP |  | CD79B |
| MAN2B2 |  | RPS28 |
| PLSCR1 |  | BANK1 |
| FLACC1 |  | IGLC1 |
| OGN |  | RPS13 |
| HHAT |  | JUNB |
| RHOJ |  | RPS23 |
| CSNK2A3 |  | RALGPS2 |
| ZNF185 |  | IGHD |
| RFXAP |  | HBA2 |
| FAM90A1 |  | MS4A1 |
| VWF |  | LTB |
|  |  | IGHM |
|  |  | IGLC2 |
|  |  | IGLC3 |
|  |  | IGKC |
|  |  | HBB |
